# Identification of Pyrrolo [2,3-*b*] Pyridine Derivatives as Novel JAK1 Inhibitors for the Treatment of Inflammatory Bowel Disease

**DOI:** 10.3390/molecules31132236

**Published:** 2026-06-25

**Authors:** Shuai Wen, Can Xiao, Li-Min Du, Jing Ji, Hong-Kai Sha, Shun-Xin-Wang Lin, Yi Mou, Hao Sun, Zheng-Yu Jiang

**Affiliations:** 1 College of Pharmacy, Taizhou University, Taizhou 225300, China; 2 Jiangsu Key Laboratory of Drug Design and Optimization, China Pharmaceutical University, Nanjing 210009, China; 3 Nanjing Linkinovo Biological Technology Research Institute, Nanjing 211100, China

**Keywords:** inflammatory bowel disease, JAK1 inhibitors, tofacitinib, scaffold growth strategy

## Abstract

Treatment of inflammatory bowel disease (IBD) remains a major medical challenge due to the lack of safe and effective therapeutic agents. JAK1 has been validated as a key therapeutic target that modulates the pathological progression of IBD. In this study, using tofacitinib as the lead compound, we adopted a scaffold growth strategy to design and synthesize a series of pyrrolo [2,3-*b*] pyridine derivatives as novel JAK1 inhibitors for the treatment of IBD. Among them, compound **15** exerted potent inhibitory activity against JAK1 with an IC_50_ value of 0.48 nM. Western blot results showed that compound **15** significantly inhibited LPS-induced STAT1/3 phosphorylation in RAW264.7 cells. In addition, **15** exhibited satisfactory metabolic stability and oral bioavailability. In the DSS-induced colitis model, **15** remarkably ameliorated inflammatory symptoms, promoted epithelial repair, and inhibited the production of pro-inflammatory cytokines such as TNF-α and IL-6. Therefore, compound **15** is regarded as a promising candidate for the treatment of IBD.

## 1. Introduction

Inflammatory bowel disease (IBD) is a chronic, recurrent inflammatory disorder of the gastrointestinal tract, mainly including Crohn’s disease (CD) and ulcerative colitis (UC) [1,2,3,4,5,6]. It is primarily characterized by recurrent intestinal inflammation, impaired intestinal epithelial barrier function and intestinal microecological dysbiosis [7,8,9,10]. Studies have demonstrated that inhibiting multiple cytokine-driven inflammatory pathways is effective for the treatment of IBD [11,12,13,14,15]. Janus kinases (JAKs) interact with signal transducers and activators of transcription (STAT) proteins and play a pivotal role in cytokine receptor signaling cascades [16,17,18]. Therefore, JAKs represent promising therapeutic targets for IBD [19,20,21,22].

JAKs constitute a small family of receptor-associated tyrosine kinases and play a crucial role in cellular responses to cytokines, consisting mainly of four members: JAK1, JAK2, JAK3 and TYK2 [9,23]. Pro-inflammatory cytokines bind to cell membrane receptors and activate JAKs, which further activate STATs via phosphorylation. Subsequently, activated STATs translocate into the nucleus to regulate the transcription of inflammation-related genes, thereby mediating and amplifying intestinal inflammatory responses [24,25,26]. In the JAK-STAT signaling pathway, JAK1 acts as a core shared molecule for multiple signaling cascades, and it is the only subtype capable of forming heterodimers with the other three JAK members and phosphorylating all STAT proteins [27,28,29].

**Figure 1:**
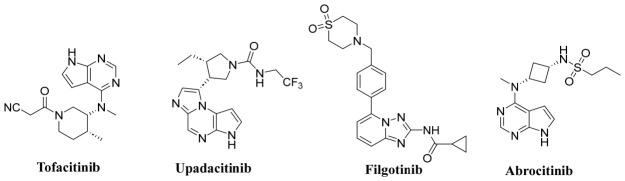
Structures of representative JAK1 inhibitors tofacitinib, upadacitinib, filgotinib and abrocitinib.

Multiple JAK1 inhibitors are currently commercially available, including tofacitinib, upadacitinib, abrocitinib and filgotinib, etc. [30,31,32,33]. (Figure 1). Notably, tofacitinib exerts a prominent therapeutic effect on the remission of UC, and is particularly applicable to patients with inadequate response to conventional therapy, making it a first-line agent for UC management in clinical practice [19,34,35]. Nevertheless, the clinical use of tofacitinib is associated with a variety of adverse reactions, such as decreased hemoglobin, reduced absolute neutrophil count, elevated total cholesterol, and increased susceptibility to various infections [36,37,38]. More importantly, the FDA has recently issued a boxed warning: tofacitinib administered at a dose of 10 mg twice daily may increase the risks of thrombosis and mortality [39,40]. Accordingly, there is an urgent need to develop novel low-toxicity tofacitinib derivatives for the treatment of IBD.

Accordingly, using tofacitinib as the lead compound, we designed and synthesized a series of pyrrolo [2,3-*b*] pyridine derivatives via the scaffold growth strategy as novel JAK1 inhibitors for the treatment of IBD. Systematic structure–activity relationship (SAR) studies revealed that compound **15** exhibited potent JAK1 inhibitory activity. Notably, in the DSS-induced acute colitis model, compound **15** markedly alleviated inflammatory symptoms, promoted epithelial repair, and suppressed the production of pro-inflammatory cytokines.

## 2. Results and Discussion

### 2.1. Molecular Design

**Figure 2:**
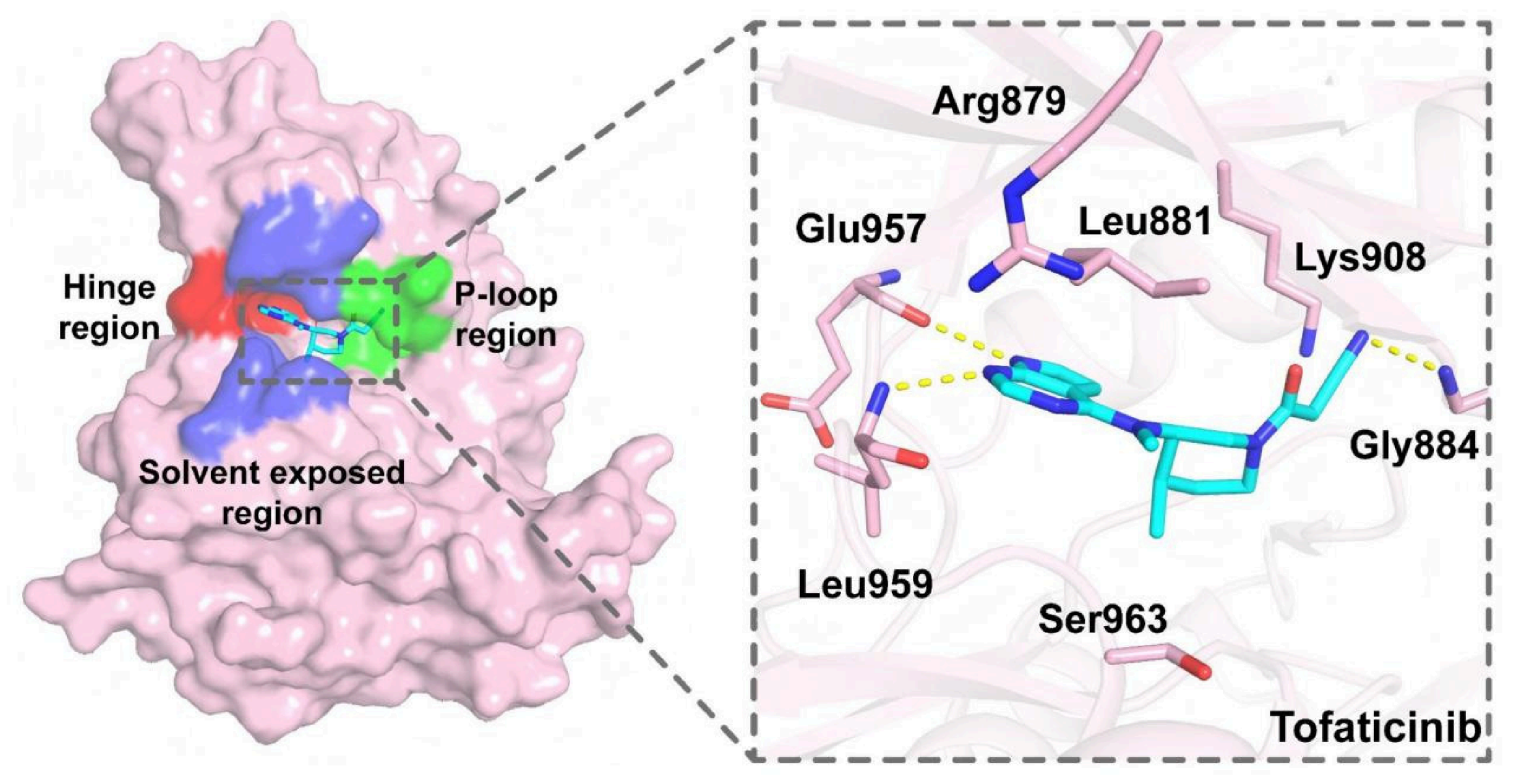
Co-crystal structure analysis of tofacitinib (cyan) and JAK1 (pink) complex (PDB: 3EYG). Yellow dashed lines represent hydrogen bonds.

We first analyzed the binding mode of tofacitinib to JAK1 using the co-crystal structure (PDB: 3EYG) (Figure 2). The pyrrolopyrimidine moiety in the tofacitinib molecule mimics the purine moiety of ATP/ADP and stably occupies the hinge region of the ATP-binding pocket of JAK1 protein. Specifically, the pyrrolopyrimidine scaffold forms hydrogen bonds with the Leu959 and Glu957 residues in the hinge region. Meanwhile, the cyanoamide-substituted piperidine moiety of tofacitinib mimics the cyclopentane and phosphate moieties of ATP/ADP. The cyanoamide fragment extends into the P-loop region, where the cyano group forms an additional hydrogen bond with the Gly884 residue. This chemical architecture and preferred conformation confer high binding affinity of tofacitinib to JAK1 protein. Furthermore, we found that the solvent-exposed region of tofacitinib is not fully occupied, leaving considerable chemical modification space that can be exploited to further improve the physicochemical properties and other relevant characteristics of the compound.

**Figure 3:**
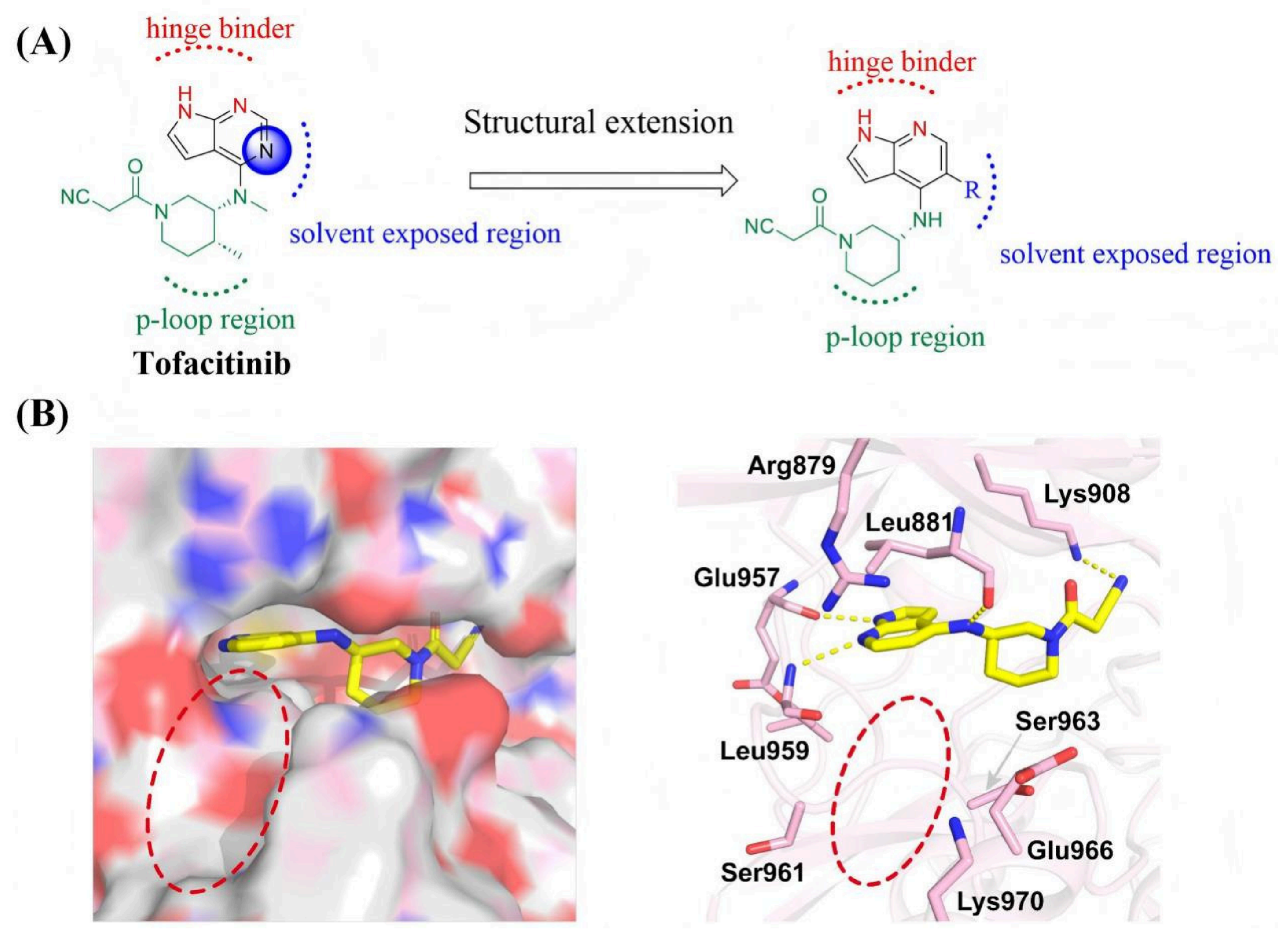
Development strategy of JAK1 inhibitors. (**A**) Rational design of target compounds and hit optimization regions. (**B**) 3D binding poses of pyrrolo [2,3-*b*] pyridine scaffold (yellow) in ATP-binding pocket of JAK1 protein (pink) (PDB: 3EYG). The red dashed line indicates the solvent-exposed region. Yellow dashed lines represent hydrogen bonds.

Building on these findings, we conducted rational structural design using tofacitinib as the lead compound. To achieve scaffold extension, we performed a C-N atom replacement in the core backbone, and designed a novel class of JAK1 inhibitors bearing a pyrrolopyridine scaffold (Figure 3A). To elucidate the interactions between this scaffold and JAK1 protein, we performed molecular docking studies, and the results are shown in Figure 3B. Docking results revealed that the pyrrolopyridine scaffold forms a stable hydrogen bond network with residues Leu959 and Glu957 in the hinge region of JAK1, and its cyanoamide-substituted piperidine moiety can well occupy the hydrophobic pocket of the protein. Furthermore, we identified a set of key amino acid residues, including Arg879, Ser961, Ser963, Glu966 and Lys970, distributed in the solvent-exposed region of JAK1. Given that introducing appropriate substituents into the solvent-exposed region could potentially establish additional interactions with these residues, we adopted the scaffold growth strategy by introducing linear moieties or bulky aromatic groups into this region to fully occupy the pocket. Aiming to form additional hydrophobic interactions and hydrogen bonds with the surrounding amino acid residues in the solvent-exposed region, we finally designed and synthesized a series of novel small-molecule JAK1 inhibitors.

### 2.2. Structure−Activity Relationship (SAR) Discussion

Next, we conducted a systematic SAR study on the R substituents of the pyridine ring, with detailed results presented in Table 1. Based on the distance characteristics between the amino acid residues surrounding the solvent-exposed region of the JAK1 protein and the molecular scaffold, we first introduced 5,6-fused heteroaromatic substituents to evaluate their effects on JAK1 inhibitory activity. Among them, compound **9** bearing a pyrazolopyridine group (IC_50_ = 3.532 ± 0.190 nM), compound **10** containing a pyrrolopyridine moiety (IC_50_ = 36.5 ± 3.694 nM), and compound **11** (IC_50_ = 90.07 ± 5.940 nM) all exhibited inferior inhibitory potency to tofacitinib. We then further investigated the impact of introducing 6,6-fused heteroaromatic substituents on biological activity. The results showed that compounds incorporating quinoline (**12**), isoquinoline (**13**), and quinazoline (**14**) moieties had similar degrees of loss in inhibitory efficacy, with IC_50_ values all falling within the range of 35–50 nM. Notably, when the solvent-exposed region was occupied by a bicyclic aromatic system, all tested compounds showed varying degrees of decreased JAK1 inhibitory activity. A plausible explanation is that a certain torsional angle formed between the pyrrolopyridine scaffold and the bicyclic structural system may increase the steric hindrance, which in turn restricts the formation of the preferred molecular conformation and weakens the complementarity and binding interaction with the ATP-binding pocket of JAK1 protein. Building on these findings, we further introduced a linear alkynylpyridine moiety at this position to obtain compound **15**. This compound displayed potent JAK1 inhibitory activity (IC_50_ = 0.48 ± 0.427 nM), with significantly improved JAK1 inhibitory potency compared to tofacitinib. This finding indicates that the linear alkynylpyridine structure can effectively adapt to the spatial constraints of the solvent-exposed region and enhance the binding interaction between the molecule and the protein. In summary, compound **15** exhibited the optimal JAK1 inhibitory activity, which defined the direction for further structural modifications in subsequent studies.

Then, we investigated the effects of different types of aminopiperidine scaffolds on JAK1 inhibitory potency. The results presented in Table 2 demonstrate that both compound **16** (IC_50_ = 10.54 ± 1.548 nM) and compound **18** (IC_50_ = 23.27 ± 1.209 nM) resulted in reduced JAK1 inhibitory activity. Furthermore, compound **17** exhibited a markedly decreased JAK1 inhibitory activity with an IC_50_ value of 61.74 ± 10.720 nM. It is hypothesized that the S-configured R_1_ group orients toward the hydrophilic side of the binding pocket and forms unfavorable interactions with the polar residues within the pocket, thereby attenuating the binding interaction between the small molecule and JAK1 protein.

### 2.3. Molecular Docking and MD Study of Compound ***9*** and ***15***

**Figure 4:**
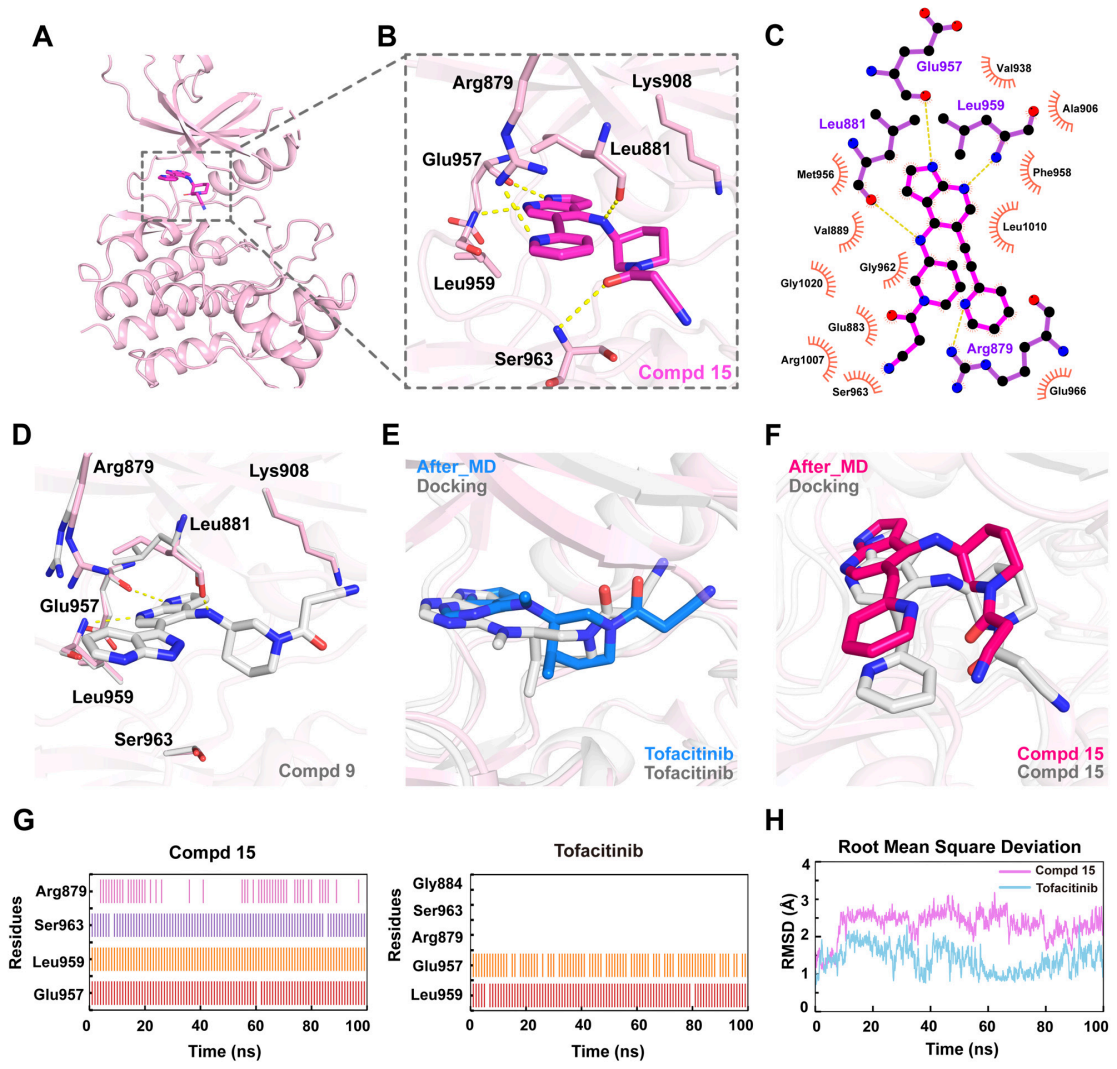
Molecular docking and molecular dynamics simulation analyses of JAK1 inhibitors. (**A**) Overall docking pose of compound **15** in the ATP-binding pocket of JAK1. (**B**) Detailed interactions between compound **15** and key residues of JAK1. (**C**) Two-dimensional interaction diagram of compound **15** in the JAK1 binding pocket. (**D**) Docking pose of compound **9** in the JAK1 ATP-binding pocket. The compound **15**-JAK1 binding model was used as a structural reference, with compound **15** omitted for clarity. (**E**) Superposition of the initial structures and representative conformations after 100 ns MD simulations for tofacitinib. (**F**) Superposition of the initial structures and representative conformations after 100 ns MD simulations for compound **15**. (**G**) Time evolution of key JAK1–ligand interactions during the 100 ns MD simulations. The trajectories were analyzed every 1 ns, and each vertical line represents one detected interaction. Arg879 indicates a π-cation interaction; the other residues indicate hydrogen bonds. (**H**) Ligand RMSD profiles of compound **15** and tofacitinib during the 100 ns MD simulations. Pink and blue indicate compound **15** and tofacitinib, respectively. Yellow dashed lines indicate hydrogen bonds or polar interactions. The figures were prepared using PyMOL 2.6.2.

To elucidate the binding mode of compound **15**, we performed molecular docking simulations using the JAK1 co-crystal structure (PDB: 3EYG) as the template (Figure 4A–C). The docking results revealed that compound **15** adopted a binding mode similar to that of tofacitinib. Its pyrrolopyridine scaffold formed a stable hydrogen bond network with the hinge region residues Glu957 and Leu959. Meanwhile, the aminopiperidine moiety occupied the polar pocket and established hydrogen bond interactions with residues Leu881 and Ser963. Furthermore, the lipophilic alkynylpyridine group introduced into the solvent-exposed region formed a hydrogen bond between its pyridine nitrogen and residue Arg879. This interaction simultaneously induced a conformational rearrangement of the compound, rendering its molecular structure better accommodated to the ATP-binding cavity of JAK1 protein. To further elucidate the reasons for the low potency of bicyclic aromatic substituted compounds, we performed molecular docking analysis on the representative compound **9** and compared its binding pose with that of compound **15** (Figure 4D). The docking results reveal that the bicyclic heteroaromatic substituent of compound **9** has a large volume and rigid conformation. It occupies a broader spatial range within the solvent-exposed region of JAK1. This region features a narrow and elongated topology due to steric constraints imposed by surrounding amino acid residues. Consequently, the bulky bicyclic moiety reduces the spatial complementarity between the ligand and the binding pocket. Further analysis indicates that compound **9** adopts an altered binding orientation relative to compound **15**. This shift prevents its cyanoamide-substituted piperidine fragment from maintaining polar interactions with Ser963. Collectively, although compound **9** can still fit into the narrow subpocket of the solvent-exposed region of JAK1, its large bicyclic substituent impedes the formation of the optimal binding conformation and disrupts the interaction network with peripheral amino acid residues. This is likely the primary reason why compounds **9**–**14** exhibit weaker inhibitory activity than compound **15**.

To further evaluate the binding stability of compound **15**, we performed 100 ns molecular dynamics (MD) simulations on compound **15** and tofacitinib. Root-mean-square deviation (RMSD) analysis of the ligands indicated that both compounds reached a relatively stable binding state throughout the simulation (Figure 4H). A comparison between the initial MD conformation and representative conformations after simulation revealed that both ligands remained within the ATP-binding pocket of JAK1 after the 100 ns simulation. No obvious dissociation or large-scale displacement was observed, indicating that both compounds could maintain basically stable binding modes (Figure 4E,F). Further interaction analysis revealed distinct differences in dynamic binding behaviors between compound **15** and tofacitinib. During molecular dynamics (MD) simulations, compound **15** stably sustained polar interactions with the main chains of Glu957 and Leu959 in the hinge region, and also formed persistent polar contacts with the hydroxyl group of Ser963. Moreover, the alkynylpyridine moiety of compound **15** extended toward the solvent-exposed side of the ATP-binding pocket, enabling the formation of relatively stable π–cation interactions with Arg879. In contrast, tofacitinib retained the canonical hinge region interactions with the main chains of Glu957 and Leu959. However, it lacks the linear extended alkynylpyridine fragment found in compound **15**, and thus failed to establish continuous interactions with Ser963 or Arg879 throughout the MD simulation. Additionally, the interaction with Gly884 observed in the initial binding pose of tofacitinib was not sustained during the dynamic simulation (Figure 4G). These results demonstrate that compound **15** not only retains the canonical binding mode with the hinge region, but also further strengthens the intermolecular interaction network on the solvent-exposed side of the ATP-binding pocket via its alkynylpyridine fragment. This additional network of interactions, particularly the persistent contacts with Ser963 and Arg879, may help improve the binding stability and potential pocket residence capacity of compound **15** within the JAK1 ATP-binding pocket. Collectively, these observations provide further structural and dynamic evidence explaining why compound **15** exhibits superior inhibitory activity relative to tofacitinib.

### 2.4. Immunoblotting Confirmed the Inhibition of ***15*** in the JAK/STAT Signaling Pathway

**Figure 5:**
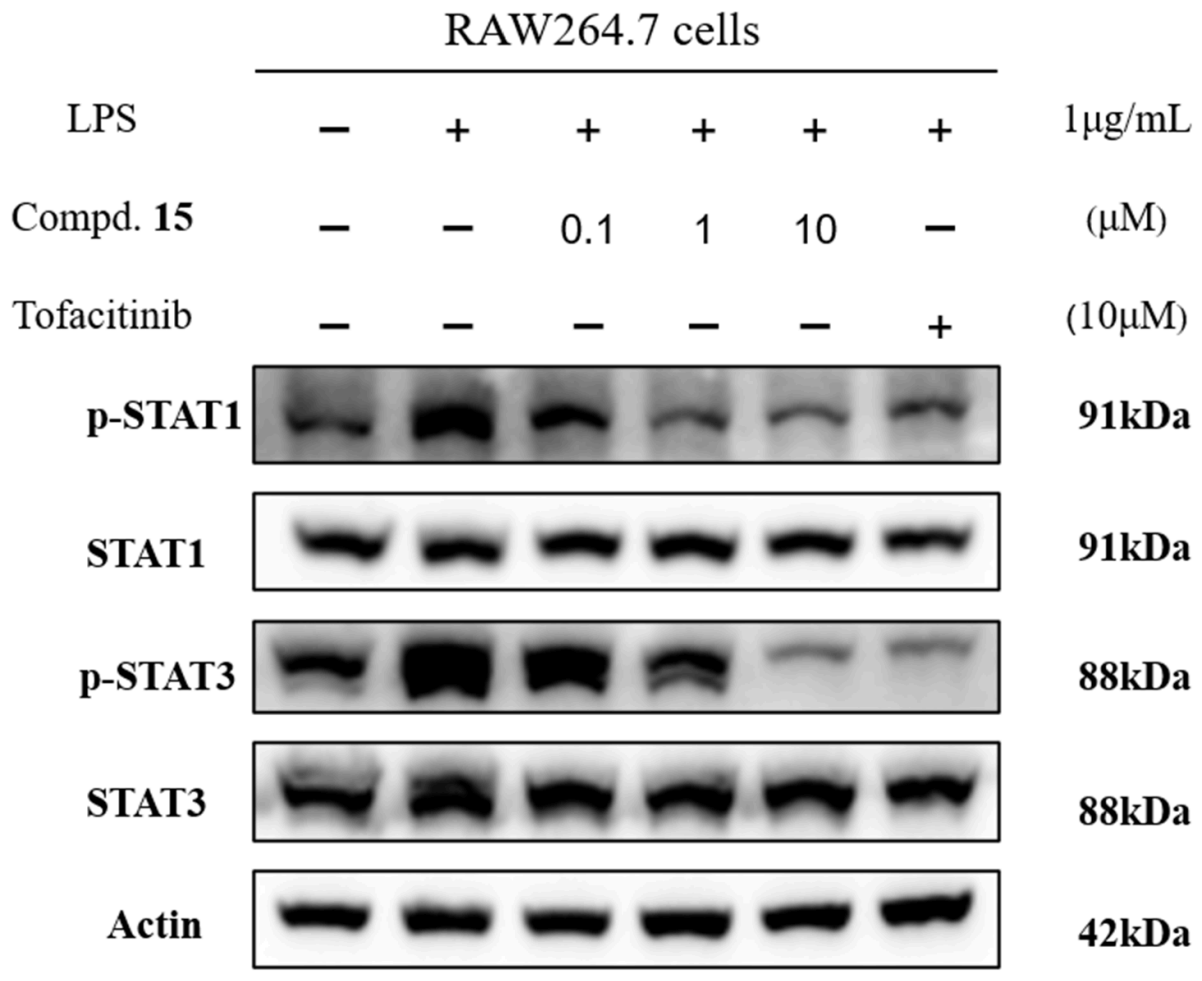
Compound **15** inhibited LPS-induced phosphorylation of STATs in vitro. Cells were stimulated with 1 μg/mL LPS, immediately followed by the addition of different concentrations of the test compounds, and co-incubated for 24 h.

JAK kinase activation is initiated by distinct cytokines, which induces JAK dimerization and mediates STAT protein phosphorylation. To verify the efficacy of compound **15**, we performed Western blot analysis in RAW264.7 cells using tofacitinib as the positive control. Upon LPS stimulation, cells were treated with different concentrations of compound **15**, and the inhibitory effects on STAT phosphorylation were examined, as shown in Figure 5. Following stimulation with 1 μg/mL LPS, compound **15** inhibited the phosphorylation levels of STAT1/3 in a dose-dependent manner. Notably, at a concentration of 10 μM, compound **15** markedly inhibited LPS-induced phosphorylation, and at this concentration, it exhibited a phosphorylation inhibitory effect comparable to that of tofacitinib. Collectively, compound **15** demonstrated favorable inhibitory activity against the JAK/STAT signaling pathway at the cellular level.

### 2.5. Drug-like Properties Evaluation of ***15***

**Figure 6:**
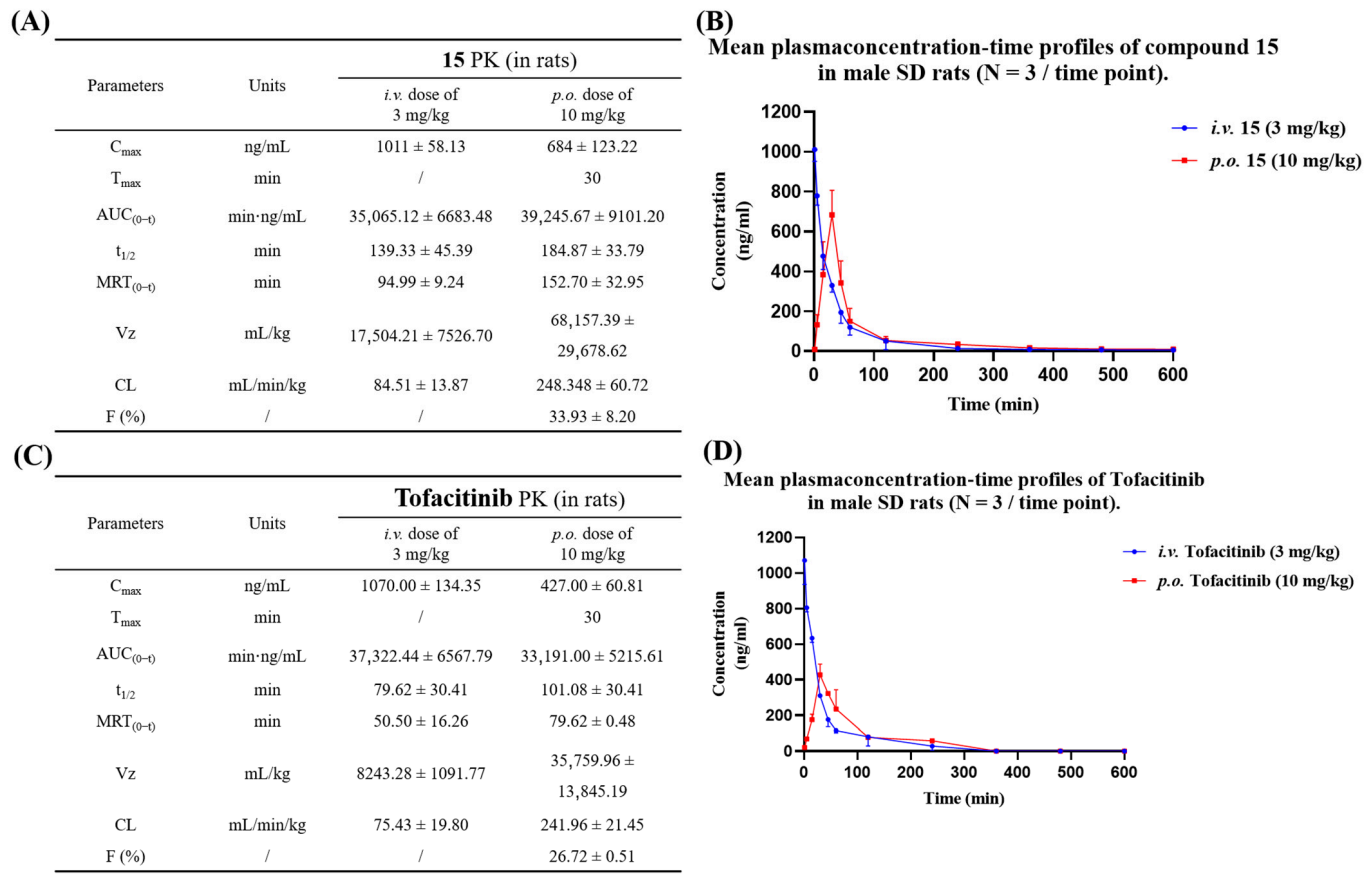
Pharmacokinetic properties of compound **15** and Tofacitinib. (**A**) In vivo PK parameters of **15** in male rats. (**B**) Concentration–time curves of **15** after intravenous injection (3 mg/kg) or oral administration (10 mg/kg) in SD rats. (**C**) In vivo PK parameters of Tofacitinib in male rats. (**D**) Concentration–time curves of Tofacitinib after intravenous injection (3 mg/kg) or oral administration (10 mg/kg) in SD rats. The values shown are the means ± SD (*n* = 3).

Given its favorable JAK1 potency, we evaluated the drug-like properties of compound **15**. As shown in Table 3, compound **15** had an aqueous solubility of 462.34 μg/mL and exhibited excellent stability in simulated gastric fluid (SGF), simulated intestinal fluid (SIF), rat plasma, and rat liver microsomes (RLMs). We further assessed the pharmacokinetic (PK) profiles of compound **15** and tofacitinib. Two compounds were respectively dissolved in a vehicle consisting of 10% DMSO, 40% PEG 400, and 50% water, and administered to SD rats via intravenous (IV: 3 mg/kg) and oral (PO: 10 mg/kg) routes. The corresponding pharmacokinetic parameters are presented in Figure 6. A comparative analysis of key pharmacokinetic (PK) parameters revealed that compound **15** exhibited higher oral bioavailability than tofacitinib under identical experimental conditions. This indicates that compound **15** has favorable oral absorption and undergoes less hepatic first-pass metabolism, so ideal plasma drug concentrations can be achieved at low oral doses. At the oral dose of 10 mg/kg, the maximum plasma concentration (C_max_) of compound **15** was 684 ng/mL, with the area under the concentration–time curve (AUC) of 39,245.67 min·ng/mL. In comparison, tofacitinib showed a C_max_ of 427 ng/mL and an AUC of 33,191.00 min·ng/mL. The higher systemic exposure of compound **15** at the same dose facilitates sustained pharmacological effects. The oral elimination half-life (T_1/2_) of compound **15** was 184.87 min, which was markedly longer than that of tofacitinib (101.08 min). A prolonged half-life corresponds to slower in vivo elimination, which is expected to reduce dosing frequency and improve patient compliance. Additionally, compound **15** displayed a moderate clearance rate and a larger apparent volume of distribution, suggesting a better potential for tissue distribution in vivo. Collectively, these findings support further in vivo evaluation of compound **15**.

### 2.6. Evaluation of the Therapeutic Effect of ***15*** in an Acute Mice Colitis Model

**Figure 7:**
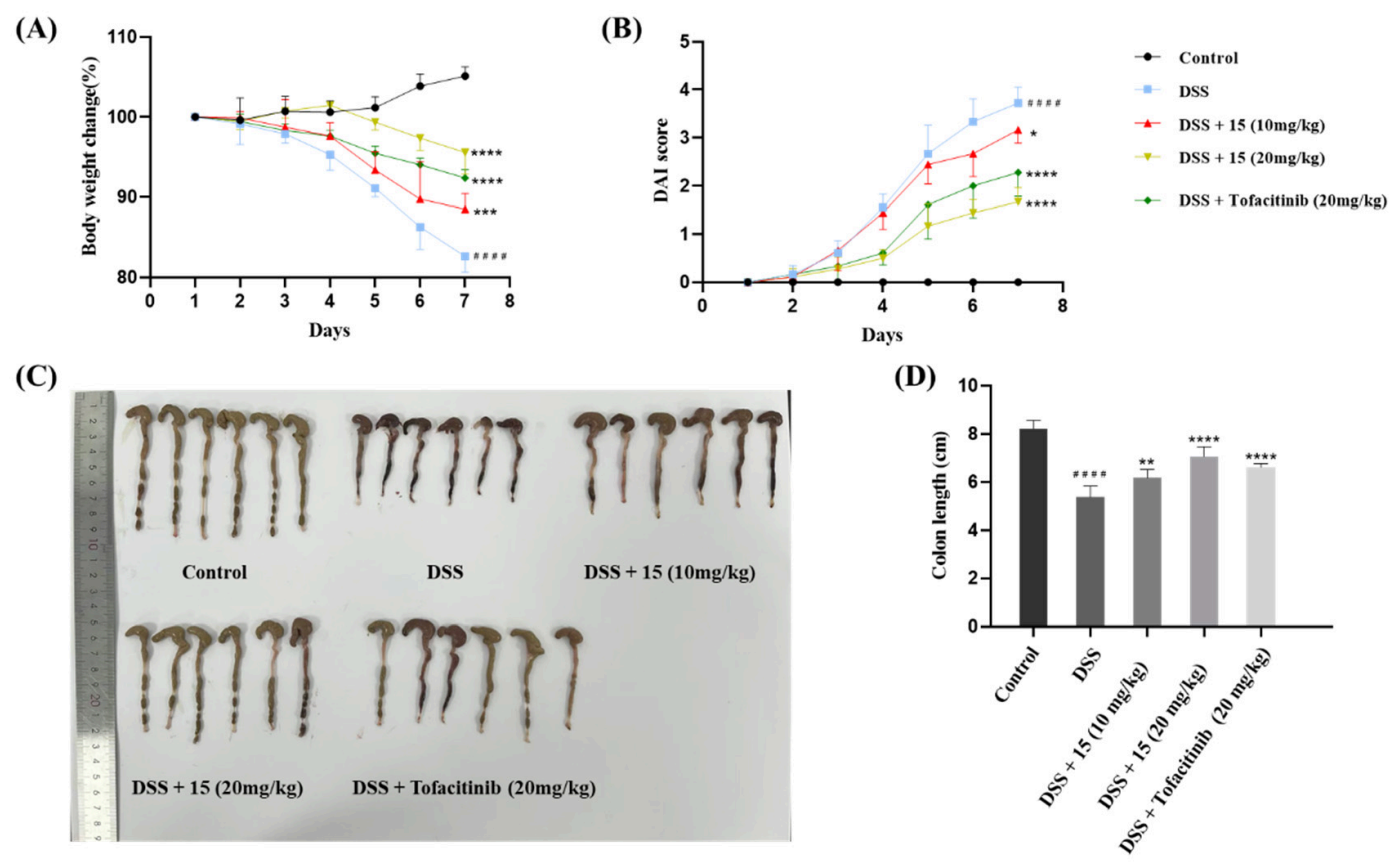
Therapeutic efficacy of compound **15** in the acute mice colitis model (*n* = 6 per group). (**A**) Changes in body weight across different treatment groups. (**B**) DAI scores in different groups. (**C**) Gross views of the colons in the different experimental groups at the end of the administration. (**D**) Colon length measurements for mice in different groups. Values in (**A**,**B**,**D**) are presented as means ± SD (*n* = 6). Statistical significance was determined by one-way ANOVA with Tukey’s multiple comparisons test: ^####^ *p* < 0.0001, vs. control group; **** *p* < 0.0001, *** *p* < 0.001, ** *p* < 0.01, * *p* < 0.05, vs. DSS group.

**Figure 8:**
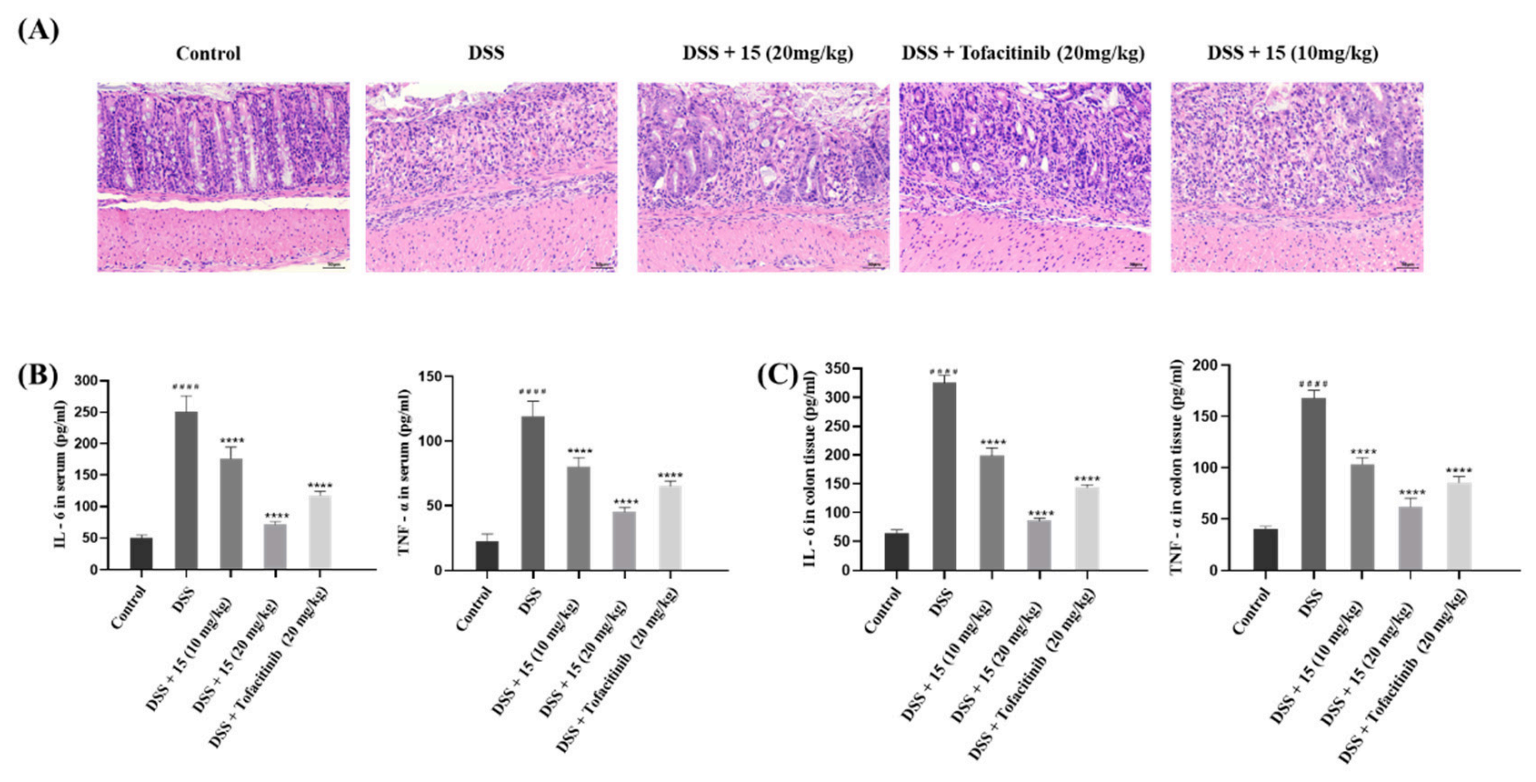
(**A**) Representative H&E staining images of colon sections (Scale bar = 50 μm). (**B**) Levels of IL-6 and TNF-α in serum. (**C**) Levels of IL-6 and TNF-α in the colon tissue. Values in (**B**,**C**) are presented as means ± SD (*n* = 6). Statistical significance was determined by one-way ANOVA with Tukey’s multiple comparisons test: ^####^ *p* < 0.0001, vs. control group; **** *p* < 0.0001, vs. DSS group.

We established dextran sulfate sodium (DSS)-induced acute ulcerative colitis mouse models to further evaluate the in vivo therapeutic efficacy of compound **15**. Acute murine colitis was induced using 3% (*w*/*v*) DSS, and tofacitinib was selected as the positive control. Tofacitinib (20 mg/kg) and compound **15** (10 mg/kg or 20 mg/kg) were administered orally once daily (qd) for 7 consecutive days. The severity of colitis was assessed by measuring body weight, disease activity index (DAI) score, and colon length. Compared with the normal control group, mice in the DSS model group developed severe colitis symptoms, manifested as significant body weight loss, elevated DAI scores, and shortened colon length (Figure 7A–D). Treatment with compound **15** significantly ameliorated these symptoms, as evidenced by attenuated body weight loss, reduced DAI scores, and preserved colon length. Its therapeutic efficacy was comparable to that of tofacitinib at the same dose (20 mg/kg). Notably, a significant dose–response relationship was observed, with the higher dose of compound **15** (20 mg/kg) exhibiting more pronounced therapeutic effects. Histopathological examination of hematoxylin and eosin (H&E)-stained colon tissue sections further revealed that compound **15** alleviated DSS-induced tissue damage and inflammatory cell infiltration (Figure 8A). Subsequently, the levels of pro-inflammatory cytokines (IL-6 and TNF-α) in colon tissues and serum were further evaluated by enzyme-linked immunosorbent assay (ELISA) (Figure 8B,C). The results showed that the levels of pro-inflammatory cytokines in colon tissues and serum were significantly elevated in the DSS model group, whereas treatment with compound **15** markedly downregulated these inflammatory markers, demonstrating potent anti-inflammatory activity. Collectively, these experimental results demonstrated that compound **15** significantly attenuates DSS-induced acute colitis symptoms in mice, indicating its promising in vivo therapeutic potential.

### 2.7. Synthesis of the Target Compounds

**Scheme 1:**
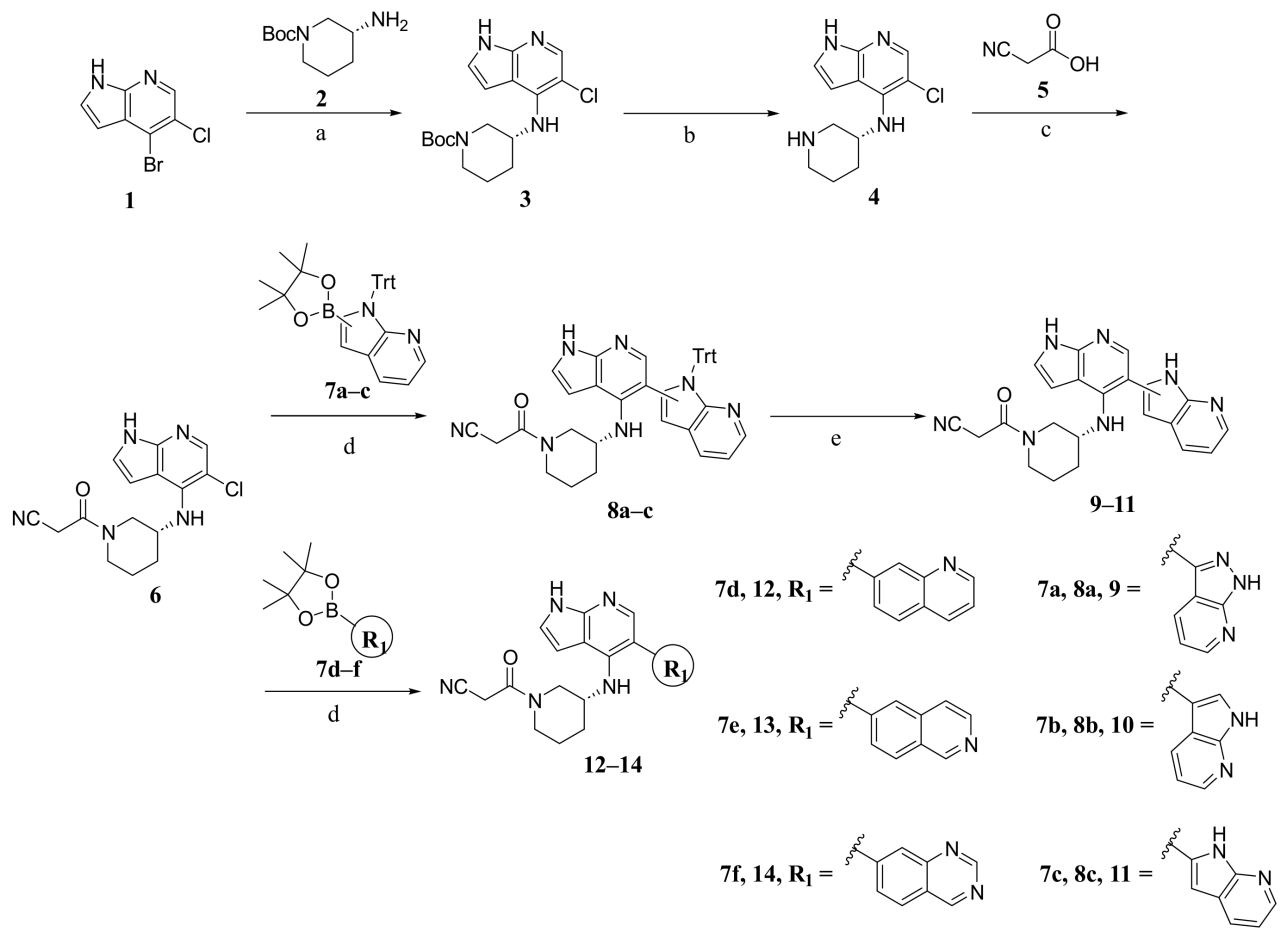
Synthetic route of compounds **9**–**14**. Reagents and conditions: (a) Pd_2_(dba)_3_, 1,3-*Bis* (2,6-diisopropylphenyl) imidazolium chloride, Sodium *tert*-butoxide, 1,4-dioxane, 100 °C, 2 h, 66%; (b) CF_3_COOH, CH_2_Cl_2_, rt, 1 h; (c) 2-cyanoacetic acid, DCC, *N*,*N*-Diisopropylethylamine, CH_2_Cl_2_, rt, 6 h, 60%; (d) XPhos Pd G_2_, Cs_2_CO_3_, 1,2-dimethoxyethane and water, 100 °C, 2 h, 75–80%; (e) CF_3_COOH, CH_2_Cl_2_, rt, 1 h, 40–60%.

**Scheme 2:**
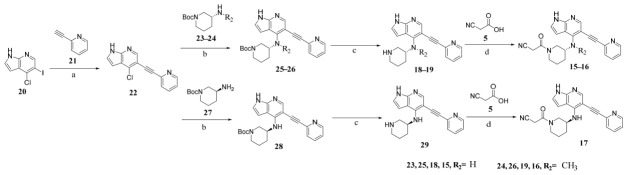
Synthetic route of compounds **15**–**18**. Reagents and conditions: (a) ethynylpyridine, (PPh_3_)_2_PdCl_2_, CuI, triethylamine, CH_3_CN, 90 °C, 4 h, 80%; (b) Pd_2_(dba)_3_, 1,3-*Bis* (2,6-diisopropylphenyl) imidazolium chloride, Sodium *tert*-butoxide, 1,4-dioxane, 100 °C, 2 h, 65%; (c) CF_3_COOH, CH_2_Cl_2_, rt, 1 h, 85%; (d) 2-cyanoacetic acid, DCC, *N*,*N*-Diisopropylethylamine, CH_2_Cl_2_, rt, 6 h, 50–60%.

The structures and synthetic routes of the target compounds are detailed in Scheme 1 and Scheme 2. The target compounds **9**–**14** were synthesized following the synthetic procedure outlined in Scheme 1. Starting from compound **1**, intermediate **3** was obtained via Buchwald–Hartwig cross-coupling reaction, followed by Boc deprotection with an appropriate amount of trifluoroacetic acid to afford intermediate **4**. Condensation of intermediate **4** with cyanoacetic acid in anhydrous dichloromethane as the solvent yielded the key intermediate **6**. Intermediate **6** was converted to intermediates **8a–c** and target compounds **12**–**14** via Suzuki cross-coupling reaction, and final deprotection with an appropriate amount of trifluoroacetic acid furnished the target compounds **9**–**11**.

Target compounds **15**–**18** were synthesized following the procedure outlined in Scheme 2. Starting from compound **20**, intermediate **22** was obtained via Sonogashira cross-coupling reaction. Compounds **25**, **26** and **28** were prepared via Buchwald–Hartwig cross-coupling reaction, followed by Boc deprotection with an appropriate amount of trifluoroacetic acid to afford intermediates **18**, **19** and **29**. Subsequent condensation with cyanoacetic acid in anhydrous dichloromethane as the solvent yielded target compounds **15**, **16** and **17**.

## 3. Experimental Section

### 3.1. Chemistry

All chemical solvents and reagents were purchased from commercial sources unless otherwise stated. Reactions were monitored by thin-layer chromatography (TLC) on 0.25 mm silica gel plates (GF-254) and visualized under UV light at wavelengths of 254 and 365 nm. Column chromatography was performed on silica gel (200–300 mesh) for the separation and purification of target compounds. Chemical structures were identified by ^1^H NMR spectroscopy, ^13^C NMR spectroscopy, and high-resolution mass spectrometry (HRMS). ^1^H NMR and ^13^C NMR spectra were recorded on a Bruker AVANCE 300 spectrometer (Bruker, Leipzig, Germany) using deuterated solvents with tetramethylsilane (TMS) as the internal standard. HRMS spectra were recorded on a Waters Q-Tof micro mass spectrometer. Compound purities were determined by high-performance liquid chromatography (HPLC) on a Shimadzu instrument (Shimadzu, Kyoto, Japan) equipped with an Inertsil C18 column (4.6 mm × 250 mm, 5 μm) using a methanol/water mobile phase at a flow rate of 1 mL/min, with detection at 254 nm. All final compounds were confirmed to have purities >95% by HPLC analysis.

#### 3.1.1. Procedure for Preparation of **22**

4-Chloro-5-(pyridin-2-ylethynyl)-1H-pyrrolo [2,3-*b*]pyridine (**22**)

To 25 mL of acetonitrile, compounds **20** (1.00 g, 3.60 mmol, 1.00 equiv) and **21** (555.12 mg, 5.93 mmol, 1.50 equiv), Pd(PPh_3_)_2_Cl_2_ (126.36 mg, 0.18 mmol, 0.05 equiv), CuI (68.40 mg, 0.36 mmol, 0.10 equiv), and triethylamine (1.18 g, 10.80 mmol, 3.00 equiv) were added. The reaction mixture was degassed and placed under an argon atmosphere, then heated to reflux at 90 °C. Upon completion of the reaction, the solvent was evaporated under reduced pressure. The residue was extracted with ethyl acetate, and the organic layer was washed sequentially with water (2 × 100 mL) and brine (1 × 100 mL), dried over anhydrous sodium sulfate, and concentrated under reduced pressure to afford the crude product. Purification by column chromatography (DCM:MeOH = 250:1, *v*/*v*) afforded 728 mg of compound **22** as a yellow solid in 80% yield. ^1^H NMR (300 MHz, DMSO-*d*_6_) *δ* 12.39 (s, 1H), 8.65 (d, *J* = 4.8 Hz, 1H), 8.52 (s, 1H), 7.91–7.86 (m, 1H), 7.76–7.67 (m, 2H), 7.48–7.43 (m, 1H), 6.63–6.59 (m, 1H). MS (ESI) *m*/*z*: 254.1 [M + H]^+^.

#### 3.1.2. Procedure for General Preparation of **3** and **25**, **26**, **28**

*tert*-butyl (*R*)-3-((5-(pyridin-2-ylethynyl)-1*H*-pyrrolo [2,3-*b*]pyridin-4-yl)amino)piperidine-1-carboxylate (**25**)

To 5 mL of dioxane, compound **22** (250.00 mg, 0.99 mmol, 1.00 equiv), 4 g (296.80 mg, 1.48 mmol, 1.50 equiv), t-BuONa (596.44 mg, 5.93 mmol, 6.00 equiv), 1,3-bis(2,6-diisopropylphenyl)imidazolium chloride (63.65 mg, 0.15 mmol, 0.15 equiv) and Pd_2_(dba)_3_ (63.78 mg, 0.07 mmol, 0.07 equiv) were added. The reaction mixture was degassed and purged with argon, then heated to reflux at 110 °C. Upon completion of the reaction, the solvent was evaporated under reduced pressure. The residue was extracted with ethyl acetate, and the organic layer was washed sequentially with water (2 × 100 mL) and brine (1 × 100 mL), dried over anhydrous sodium sulfate, and concentrated under reduced pressure. Purification by column chromatography (DCM:MeOH = 200:1, *v*/*v*) afforded 268 mg of compound **25** as a yellow solid in 65% yield. ^1^H NMR (300 MHz, DMSO-*d*_6_) *δ* 11.64 (s, 1H), 8.59 (dd, *J* = 5.0, 1.7 Hz, 1H), 8.08 (s, 1H), 7.90–7.70 (m, 1H), 7.60 (d, *J* = 7.8 Hz, 1H), 7.40–7.32 (m, 1H), 7.26 (t, *J* = 2.9 Hz, 1H), 6.66 (s, 1H), 5.83 (d, *J* = 8.5 Hz, 1H), 4.20–4.28 (m, 1H), 3.95–3.89 (m, 1H), 3.57 (d, *J* = 12.8 Hz, 1H), 3.26–3.07 (m, 2H), 2.15–1.97 (m, 1H), 1.78–1.65 (m, 2H), 1.60–1.52 (m, 1H), 1.24 (s, 9H). MS (ESI) *m*/*z*: 418.2 [M + H]^+^.

*tert*-butyl (*R*)-3-(methyl(5-(pyridin-2-ylethynyl)-1*H*-pyrrolo [2,3-*b*]pyridin-4-yl)amino)piperidine-1-carboxylate (**26**)

Compound **26** was synthesized according to the same procedure as **25** using **22** and **24**. The crude product is used directly in the next step without purification.

(*S*)-3-((5-(pyridin-2-ylethynyl)-1*H*-pyrrolo [2,3-*b*]pyridin-4-yl)amino)piperidine-1-carboxylate (**28**)

Compound **28** was synthesized according to the same procedure as **25** using **22** and **27**. The crude product is used directly in the next step without purification.

*tert*-butyl (*R*)-3-((5-chloro-1*H*-pyrrolo [2,3-*b*]pyridin-4-yl)amino)piperidine-1-carboxylate (**3**)

Compound **3** was synthesized according to the same procedure as **25** using **1** and **2**. yield: 66%. ^1^H NMR (300 MHz, DMSO-*d*_6_) *δ* 11.51 (s, 1H), 7.89 (s, 1H), 7.26 (t, *J* = 3.0 Hz, 1H), 6.56 (d, *J* = 3.5 Hz, 1H), 5.46 (d, *J* = 8.3 Hz, 1H), 4.08–4.03 (m, 1H), 3.87 (d, *J* = 13.0 Hz, 1H), 3.57 (dd, *J* = 12.5, 5.7 Hz, 1H), 3.22 (s, 1H), 3.13–3.07 (m, 1H), 2.02–1.59 (m, 4H), 1.30 (s, 9H). MS (ESI) *m*/*z*: 351.1 [M + H]^+^.

#### 3.1.3. Procedure for General Preparation of **4**, **18**, **19** and **29**

(*R*)-*N*-(piperidin-3-yl)-5-(pyridin-2-ylethynyl)-1*H*-pyrrolo [2,3-*b*]pyridin-4-amine (**18**)

To a mixture of compound **25** (200.00 mg, 0.63 mmol, 1.00 equiv) and dichloromethane (10 mL), 5 mL of trifluoroacetic acid (TFA) at room temperature was added. The reaction mixture was stirred at room temperature for 2 h. Upon completion of the reaction, the solvent was evaporated under reduced pressure. The residue was extracted with ethyl acetate, and the organic layer was washed sequentially with saturated sodium bicarbonate solution (2 × 100 mL) and brine (1 × 100 mL), dried over anhydrous sodium sulfate, and concentrated under reduced pressure to afford the crude product. Purification by column chromatography (DCM:MeOH = 25:1, *v*/*v*) afforded 170 mg of compound **18** as a white solid in 85% yield. ^1^H NMR (300 MHz, DMSO-*d*_6_) *δ* 11.61 (s, 1H), 8.59 (s, 1H), 8.05 (s, 1H), 7.83 (t, *J* = 7.7 Hz, 1H), 7.69 (d, *J* = 7.9 Hz, 1H), 7.36 (s, 1H), 7.23 (s, 1H), 6.65 (s, 1H), 6.22 (d, *J* = 8.7 Hz, 1H), 4.29 (s, 1H), 3.14 (d, *J* = 11.5 Hz, 1H), 2.82 (s, 2H), 2.72 (s, 1H), 1.92 (s, 1H), 1.70–1.54 (m, 4H); HRMS (ESI): calcd for C_19_H_19_N_5_, [M + H]^+^ 318.1713, found 318.1719; HPLC (MeOH:H_2_O with 0.1% NH_3_ = 80:20) t_R_ = 5.07 min, 98.01%.

(*R*)-*N*-methyl-*N*-(piperidin-3-yl)-5-(pyridin-2-ylethynyl)-1*H*-pyrrolo [2,3-*b*]pyridin-4-amine (**19**)

Compound **19** was synthesized according to the same procedure as **18** using **26**. The crude product is used directly in the next step without purification.

(*S*)-*N*-(piperidin-3-yl)-5-(pyridin-2-ylethynyl)-1*H*-pyrrolo [2,3-*b*]pyridin-4-amine (**29**)

**29** was synthesized according to the same procedure as **18** using **28**. The crude product is used directly in the next step without purification.

(*R*)-5-chloro-*N*-(piperidin-3-yl)-1*H*-pyrrolo [2,3-*b*]pyridin-4-amine (**4**)

Compound **4** was synthesized according to the same procedure as **18** using **3**. The crude product is used directly in the next step without purification.

#### 3.1.4. Procedure for General Preparation of **6**, **15**, **16** and **17**

(*R*)-3-oxo-3-(3-((5-(pyridin-2-ylethynyl)-1*H*-pyrrolo [2,3-*b*] pyridin-4-yl) amino) piperidin-1-yl) propanenitrile (**15**)

To 10 mL of dichloromethane compound **18** (150.00 mg, 0.47 mmol, 1.00 equiv), compound **5** (60.56 mg, 0.71 mmol, 1.50 equiv), and DCC (205.66 mg, 0.94 mmol, 2.00 equiv) were added. The reaction mixture was degassed and purged with nitrogen, then stirred at 40 °C for 2 h. Upon completion of the reaction, the solvent was evaporated under reduced pressure. The residue was extracted with ethyl acetate, and the organic layer was washed sequentially with saturated sodium bicarbonate solution (2 × 100 mL) and brine (1 × 100 mL), dried over anhydrous sodium sulfate, and concentrated under reduced pressure to afford the crude product. Purification by column chromatography (DCM:MeOH = 75:1, *v*/*v*) afforded 108 mg of compound **15** as a yellow solid in 60% yield. ^1^H NMR (300 MHz, DMSO-*d*_6_) *δ* 11.64 (s, 1H), 8.63–8.55 (m, 1H), 8.07 (s, 1H), 7.84 (t, *J* = 7.6 Hz, 1H), 7.69–7.56 (m, 1H), 7.42–7.31 (m, 1H), 7.26 (s, 1H), 6.70 (d, *J* = 11.3 Hz, 1H), 5.92–5.86 (m, 1H), 4.45–4.32 (m, 1H), 4.24 (s, 1H), 4.12 (s, 1H), 4.03–3.76 (m, 1H), 3.52 (d, *J* = 11.6 Hz, 1H), 3.20 (d, *J* = 11.5 Hz, 1H), 3.14–2.89 (m, 1H), 2.10 (s, 1H), 1.74 (s, 3H); ^13^C NMR (75 MHz, DMSO-*d*_6_) *δ* 161.66, 149.79, 149.02, 147.47, 147.09, 142.79, 136.34, 126.27, 122.54, 115.95, 104.44, 99.85, 94.64, 92.93, 85.60, 50.17, 48.75, 46.80, 45.38, 30.29, 24.75, 22.97; HRMS (ESI): calcd for C_22_H_20_N_6_O, [M + H]^+^ 385.1771, found 385.1782; HPLC (MeOH:H_2_O with 0.1% NH_3_ = 80:20) t_R_ = 5.32 min, 98.86%.

(*R*)-3-(3-(methyl(5-(pyridin-2-ylethynyl)-1*H*-pyrrolo [2,3-*b*] pyridin-4-yl) amino) piperidin-1-yl)-3-oxopropanenitrile (**16**)

Compound **16** was synthesized according to the same procedure as **15** using **19** and **5**. Yellow solid (yield: 56%). ^1^H NMR (300 MHz, DMSO-*d*_6_) δ 11.77 (s, 1H), 8.58 (d, *J* = 4.8 Hz, 1H), 8.18 (d, *J* = 6.0 Hz, 1H), 7.82 (t, *J* = 7.5 Hz, 1H), 7.57 (t, *J* = 6.0 Hz, 1H), 7.38–7.32 (m, 2H), 6.63 (s, 1H), 4.33–4.28 (m, 1H), 4.07–4.01 (m, 2H), 3.99–3.89 (m, 1H), 3.69–3.51 (m, 1H), 3.24 (s, 3H), 3.09–2.89 (m, 2H), 2.15 (s, 1H), 2.01 (d, *J* = 13.1 Hz, 1H), 1.75 (d, *J* = 12.7 Hz, 1H), 1.53 (d, *J* = 13.0 Hz, 1H); HRMS (ESI): calcd for C_23_H_22_N_6_O, [M + H]^+^ 399.1928, found 399.1935; HPLC (MeOH:H_2_O with 0.1% NH_3_ = 80:20) t_R_ = 5.40 min, 96.55%.

(*S*)-3-oxo-3-(3-((5-(pyridin-2-ylethynyl)-1*H*-pyrrolo [2,3-*b*] pyridin-4-yl) amino) piperidin-1-yl) propanenitrile (**17**)

Compound **17** was synthesized according to the same procedure as **15** using **29** and **5**. Yellow solid (yield: 50%). ^1^H NMR (300 MHz, DMSO-*d*_6_) *δ* 11.65 (s, 1H), 8.59 (d, *J* = 4.7 Hz, 1H), 8.08 (d, *J* = 3.5 Hz, 1H), 7.84 (t, *J* = 7.9 Hz, 1H), 7.70–7.55 (m, 1H), 7.37 (t, *J* = 6.2 Hz, 1H), 7.26 (s, 1H), 6.70 (d, *J* = 10.9 Hz, 1H), 5.95–5.89 (m, 1H), 4.50–4.24 (m, 1H), 4.15–3.98 (m, 2H), 3.85–3.45 (m, 2H), 3.25–2.85 (m, 2H), 2.10 (s, 1H), 1.63 (s, 3H); HRMS (ESI): calcd for C_22_H_20_N_6_O, [M + H]^+^ 385.1771, found 385.1769; HPLC (MeOH:H_2_O with 0.1% NH_3_ = 80:20) t_R_ = 6.67 min, 99.15%.

(*R*)-3-(3-((5-chloro-1*H*-pyrrolo [2,3-*b*] pyridin-4-yl) amino) piperidin-1-yl)-3-oxopropanenitrile (**6**)

Compound **6** was synthesized according to the same procedure as **15** using **4** and **5**. yield: 60%. ^1^H NMR (300 MHz, DMSO-*d*_6_) *δ* 11.51 (s, 1H), 7.89 (d, *J* = 2.6 Hz, 1H), 7.26 (t, *J* = 2.9 Hz, 1H), 6.66–6.56 (m, 1H), 5.59–5.51 (m, 1H), 4.39 (dd, *J* = 12.1, 3.2 Hz, 1H), 4.19–3.98 (m, 2H), 3.99–3.95 (m, 1H), 3.79–3.51 (m, 1H), 3.24–3.01 (m, 1H), 3.00–2.67 (m, 1H), 2.04 (d, *J* = 12.7 Hz, 1H), 1.76–1.66 (m, 3H). MS (ESI) *m/z*: 318.1 [M + H]^+^.

#### 3.1.5. Procedure for General Preparation of **9**–**11**

(*R*)-3-(3-((5-(1*H*-pyrazolo [3,4-*b*] pyridin-3-yl)-1*H*-pyrrolo [2,3-*b*] pyridin-4-yl) amino) piperidin-1-yl)-3-oxopropanenitrile (**9**)

To 5 mL of a mixed solvent of 1,2-dimethoxyethane and water, compound **6** (100.00 mg, 0.32 mmol, 1.00 equiv), compound **7a** (77.12 mg, 0.32 mmol, 1.00 equiv), Cs_2_CO_3_ (208.33 mg, 0.64 mmol, 2.00 equiv), and XphosPdG2 (24.35 mg, 0.03 mmol, 0.10 equiv) were added. The reaction mixture was degassed and purged with argon, then heated to reflux at 100 °C. Upon completion of the reaction, the solvent was evaporated under reduced pressure. The residue was extracted with ethyl acetate, and the organic layer was washed sequentially with water (2 × 100 mL) and brine (1 × 100 mL), dried over anhydrous sodium sulfate, and concentrated under reduced pressure. Subsequently, 5 mL of dichloromethane and 0.5 mL of trifluoroacetic acid were added to the residue, and the mixture was stirred at room temperature for 2 h. Upon completion of the reaction, the solvent was evaporated under reduced pressure. The residue was extracted with ethyl acetate, and the organic layer was washed sequentially with saturated aqueous sodium bicarbonate solution (2 × 100 mL) and brine (1 × 100 mL), dried over anhydrous sodium sulfate, and concentrated under reduced pressure to afford the crude product. Purification by column chromatography (DCM:MeOH = 70:1, *v*/*v*) afforded 76 mg of compound **9** as a yellow solid in 60% yield. ^1^H NMR (300 MHz, DMSO-*d*_6_) *δ* 13.79 (d, *J* = 9.4 Hz, 1H), 11.52 (s, 1H), 8.60 (d, *J* = 4.4 Hz, 1H), 8.50 (d, *J* = 7.6 Hz, 2H), 8.21 (d, *J* = 8.3 Hz, 1H), 7.27 (s, 2H), 6.73 (s, 1H), 4.31 (d, *J* = 12.4 Hz, 1H), 4.10 (t, *J* = 14.8 Hz, 2H), 3.92–3.70 (m, 1H), 3.69–3.48 (m, 1H), 3.25–3.02 (m, 2H), 2.12 (s, 1H), 1.69 (d, *J* = 35.6 Hz, 3H); ^13^C NMR (75 MHz, DMSO-*d*_6_) *δ* 162.11, 152.54, 150.47, 144.37, 143.64, 136.39, 130.13, 128.57, 127.62, 120.90, 116.69, 114.17, 106.72, 100.48, 50.83, 50.20, 47.67, 46.10, 30.99, 25.43, 24.00; HRMS (ESI): calcd for C_21_H_20_N_8_O, [M + H]^+^ 401.1833, found 401.1835; HPLC (MeOH:H_2_O with 0.1% NH_3_ = 80:20) t_R_ = 4.74 min, 99.86%.

(*R*)-3-(3-(1*H*,1′*H*-[3,5′-bipyrrolo [2,3-*b*] pyridine]-4′-ylamino) piperidin-1-yl)-3-oxopropanenitrile (**10**)

Compound **10** was synthesized according to the same procedure as **9** using **6** and **7b**. Yellow solid (yield: 55%). ^1^H NMR (300 MHz, DMSO-*d*_6_) *δ* 11.89 (t, *J* = 4.9 Hz, 1H), 11.38 (s, 1H), 8.28 (d, *J* = 4.6 Hz, 1H), 7.81 (d, *J* = 9.3 Hz, 1H), 7.74 (d, *J* = 8.3 Hz, 1H), 7.55 (d, *J* = 2.5 Hz, 1H), 7.24 (d, *J* = 4.1 Hz, 1H), 7.15–7.05 (m, 1H), 6.65 (dd, *J* = 13.4, 13.6 Hz, 1H), 5.05–4.89 (m, 1H), 4.41–4.06 (m, 1H), 4.03–3.89 (m, 2H), 3.87–3.71 (m, 1H), 3.66–3.42 (m, 1H), 2.94 (s, 1H), 2.72 (t, *J* = 11.2 Hz, 1H), 2.01–1.92 (m, 1H), 1.54 (s, 2H), 1.37 (s, 1H); ^13^C NMR (75 MHz, DMSO-*d*_6_) *δ* 162.04, 149.28, 145.33, 143.42, 127.82, 125.63, 122.94, 119.40, 116.71, 115.98, 109.63, 107.10, 106.73, 106.12, 100.28, 51.00, 49.88, 47.90, 46.00, 31.46, 25.43, 24.18; HRMS (ESI): calcd for C_22_H_21_N_7_O, [M + H]^+^ 400.1880, found 400.1881; HPLC (MeOH:H_2_O with 0.1% NH_3_ = 80:20) t_R_ = 4.54 min, 99.76%.

(*R*)-3-(3-(1*H*,1′*H*-[2,5′-bipyrrolo [2,3-*b*] pyridine]-4′-ylamino) piperidin-1-yl)-3-oxopropanenitrile (**11**)

Compound **11** was synthesized according to the same procedure as **9** using **6** and **7c**. Black solid (yield: 40%). ^1^H NMR (300 MHz, DMSO-*d*_6_) *δ* 11.90 (s, 1H), 11.48 (s, 1H), 8.19 (d, *J* = 4.6 Hz, 1H), 8.01 (s, 1H), 7.94 (d, *J* = 7.5 Hz, 1H), 7.26 (s, 1H), 7.13–7.02 (m, 1H), 6.68 (d, *J* = 13.3 Hz, 1H), 6.58 (s, 1H), 5.67 (t, *J* = 10.0 Hz, 1H), 4.27 (d, *J* = 12.7 Hz, 1H), 4.06 (s, 2H), 3.92 (s, 1H), 3.59 (dd, *J* = 13.5, 13.6 Hz, 1H), 3.14–2.84 (m, 2H), 2.08–1.96 (m, 1H), 1.61 (s, 3H); ^13^C NMR (75 MHz, DMSO-*d*_6_) *δ* 162.22, 150.25, 149.57, 144.98, 142.58, 136.05, 127.76, 123.22, 121.43, 116.72, 116.25, 105.81, 105.53, 100.77, 99.07, 50.94, 49.74, 47.74, 46.12, 31.10, 25.47, 23.85; HRMS (ESI): calcd for C_22_H_21_N_7_O, [M + H]^+^ 400.1914, found 400.1901; HPLC (MeOH:H_2_O with 0.1% NH_3_ = 80:20) t_R_ = 4.74 min, 99.07%.

#### 3.1.6. Procedure for General Preparation of **12**–**14**

(*R*)-3-oxo-3-(3-((5-(quinolin-7-yl)-1*H*-pyrrolo [2,3-*b*] pyridin-4-yl) amino) piperidin-1-yl) propanenitrile (**12**)

To 5 mL of a mixed solvent of 1,2-dimethoxyethane and water, compound **6** (100.00 mg, 0.32 mmol, 1.00 equiv), compound **7d** (80.08 mg, 0.32 mmol, 1.00 equiv), Cs_2_CO_3_ (208.32 mg, 0.64 mmol, 2.00 equiv), and XphosPdG2 (24.12 mg, 0.03 mmol, 0.10 equiv) were added. The reaction mixture was degassed and purged with argon, then heated to reflux at 100 °C. Upon completion of the reaction, the solvent was evaporated under reduced pressure. The residue was extracted with ethyl acetate, and the organic layer was washed sequentially with water (2 × 100 mL) and brine (1 × 100 mL), dried over anhydrous sodium sulfate, and concentrated under reduced pressure. Purification by column chromatography (DCM:MeOH = 90:1, *v*/*v*) afforded 104 mg of compound **12** as a yellow solid in 80% yield. ^1^H NMR (300 MHz, DMSO-*d*_6_) *δ* 11.45 (s, 1H), 8.94 (s, 1H), 8.41 (d, *J* = 8.2 Hz, 1H), 8.06–8.02 (m, 2H), 7.83 (s, 1H), 7.70–7.64 (m, 1H), 7.55 (d, *J* = 8.4 Hz, 1H), 7.28 (s, 1H), 6.67 (d, *J* = 8.4 Hz, 1H), 5.18–5.10 (m, 1H), 4.25–4.08 (m, 1H), 4.03 (d, *J* = 5.6 Hz, 1H), 3.93 (d, *J* = 8.3 Hz, 1H), 3.83 (s, 1H), 3.66–3.42 (m, 1H), 3.10–2.74 (m, 2H), 1.98 (s, 1H), 1.54 (s, 3H); ^13^C NMR (75 MHz, DMSO-*d*_6_) *δ* 162.16, 151.33, 150.40, 148.68, 145.09, 144.32, 139.69, 136.34, 129.54, 129.25, 127.31, 123.31, 121.89, 116.67, 114.23, 106.69, 100.47, 50.86, 50.06, 47.63, 46.09, 30.93, 25.49, 23.88; HRMS (ESI): calcd for C_24_H_22_N_6_O, [M + H]^+^ 411.1928, found 411.1926; HPLC (MeOH:H_2_O with 0.1% NH_3_ = 80:20) t_R_ = 4.70 min, 99.55%.

(*R*)-3-(3-((5-(isoquinolin-6-yl)-1*H*-pyrrolo [2,3-*b*] pyridin-4-yl) amino) piperidin-1-yl)-3-oxopropanenitrile (**13**)

Compound **13** was synthesized according to the same procedure as **12** using **6** and **7e**. Yellow solid (yield: 75%). ^1^H NMR (300 MHz, DMSO-*d*_6_) *δ* 11.45 (s, 1H), 9.34 (d, *J* = 2.9 Hz, 1H), 8.52 (d, *J* = 5.7 Hz, 1H), 8.19 (d, *J* = 8.5 Hz, 1H), 7.97 (d, *J* = 5.7 Hz, 1H), 7.87 (d, *J* = 5.8 Hz, 1H), 7.83 (s, 1H), 7.75 (t, *J* = 8.3 Hz, 1H), 7.27 (s, 1H), 6.72–6.60 (m, 1H), 5.18–5.13 (m, 1H), 4.20 (d, *J* = 12.9 Hz, 1H), 4.01 (d, *J* = 5.3 Hz, 1H), 3.94–3.80 (m, 2H), 3.55–3.50 (m, 1H), 3.04–2.72 (m, 2H), 1.97 (d, *J* = 11.3 Hz, 1H), 1.55–1.45 (m, 3H); ^13^C NMR (75 MHz, DMSO-*d*_6_) *δ* 162.11, 152.54, 150.47, 145.21, 144.37, 143.64, 140.82, 136.39, 130.13, 128.57, 127.18, 123.31, 120.90, 116.69, 114.17, 106.72, 100.48, 50.83, 50.20, 47.67, 46.10, 30.99, 25.43, 24.00; HRMS (ESI): calcd for C_24_H_22_N_6_O, [M + H]^+^ 411.1928, found 411.1931; HPLC (MeOH:H_2_O with 0.1% NH_3_ = 80:20) t_R_ = 4.53 min, 99.78%.

(*R*)-3-(3-((5-(isoquinolin-6-yl)-1*H*-pyrrolo [2,3-*b*] pyridin-4-yl) amino) piperidin-1-yl)-3-oxopropanenitrile (**14**)

Compound **14** was synthesized according to the same procedure as **12** using **6** and **7f**. Yellow solid (yield: 78%). ^1^H NMR (300 MHz, DMSO-*d*_6_) *δ* 11.50 (s, 1H), 9.62 (d, *J* = 2.7 Hz, 1H), 9.31 (s, 1H), 8.22 (d, *J* = 8.4 Hz, 1H), 8.00 (d, *J* = 9.9 Hz, 1H), 7.88–7.81 (m, 2H), 7.28 (s, 1H), 6.68 (d, *J* = 14.6 Hz, 1H), 5.36–5.30 (m, 1H), 4.25–4.08 (m, 1H), 4.07–3.94 (m, 2H), 3.87 (s, 1H), 3.56–3.52 (m, 1H), 3.07–2.72 (m, 2H), 1.99 (s, 1H), 1.65–1.45 (m, 3H); ^13^C NMR (75 MHz, DMSO-*d*_6_) *δ* 162.16, 160.66, 155.84, 150.43, 145.43, 145.27, 144.45, 130.87, 128.71, 127.96, 124.06, 123.38, 116.66, 113.71, 106.73, 100.65, 50.82, 50.25, 47.62, 46.15, 30.92, 25.47, 23.96; HRMS (ESI): calcd for C_23_H_21_N_7_O, [M + H]^+^ 412.1880, found 412.1882; HPLC (MeOH:H_2_O with 0.1% NH_3_ = 80:20) t_R_ = 7.28 min, 93.18%.

### 3.2. JAK1 Inhibition Assay

The inhibitory activity of compounds against JAK1 kinase was performed by Beijing Aisiyipu Bio-technology Co. Ltd. (Beijing, China). The inhibitory activity assay of JAK1 was conducted using the ADP-Glo™ Kinase Assay. Prepare 2× ATP/Substrate solution (2 μM concentration) and 2× kinase solution (25 nM concentration) with kinase reaction buffer. A total of 100 nL of **15** dilutions was transferred to a 384-well assay plate using an Echo 655 liquid handler (Beckman Coulter Life Sciences, Indianapolis, IN, USA). After centrifugation, 4 μL of 2× kinase solution was added, and the plate was centrifuged at 1000 rpm for 1 min and incubated at 25 °C for 10 min. The reaction was initiated by adding 4 μL of 2× ATP/substrate solution, followed by centrifugation at 1000 rpm for 1 min and incubation at 25 °C for 60 min. A total of 4 μL of ADP-Glo reagent was then added to terminate the reaction. After centrifugation at 1000 rpm for 1 min, the plate was incubated at 25 °C for 40 min. Subsequently, 8 μL of kinase detection reagent was added, and the plate was centrifuged at 1000 rpm for 1 min and incubated at 25 °C for a further 40 min. Luminescence signals were recorded using a multimode microplate reader (Tecan, Männedorf, Switzerland).

### 3.3. Molecular Docking and MD

The X-ray crystal structure of JAK1 (3EYG) was downloaded from the Protein Data Bank (http://www.rcsb.org). Molecular docking was carried out using the extra precision (XP) mode of Glide in the Schrödinger Suite. Protein preparation was performed with the Protein Preparation Wizard (default settings), involving hydrogen addition, water removal, generation of het states via Epik, and subsequent structure optimization and minimization. The binding pocket was defined using the lattice generation tool with default parameters. Ligand structures were prepared using LigPrep v3.6 within Maestro 12.1. Docking between JAK1 and the prepared ligands was performed with the Glide docking wizard. Binding modes were analyzed using PyMOL.

Molecular dynamics simulations were performed to evaluate the dynamic binding stability of compound **15** and tofacitinib in complex with JAK1. The initial structure of the JAK1–tofacitinib complex was obtained from the co-crystal structure of JAK1 with tofacitinib (PDB ID: 3EYG), whereas the initial structure of the JAK1–compound **15** complex was derived from the molecular docking model. The protein was treated with the CHARMM36 force field, and ligand parameters were generated using CGenFF v3.0.1. All complexes were placed in a 10 × 10 × 10 nm^3^ cubic periodic water box, solvated with the TIP3P water model, and neutralized by adding Na^+^ or Cl^−^ ions. After energy minimization, the systems were equilibrated sequentially under NVT and NPT ensembles. Subsequently, each system was subjected to a 100 ns production MD simulation. Long-range electrostatic interactions were treated using the PME method, and bonds involving hydrogen atoms were constrained using the LINCS algorithm with a 2 fs time step. After the simulations, ligand RMSD values were calculated, and the frequencies of key protein–ligand interactions were analyzed. For the interaction timeline analysis, one frame was extracted every 1 ns from each trajectory.

### 3.4. Cell Culture and Reagents

Lipopolysaccharide (LPS) was purchased from Biyuntian Biotechnology (Shanghai, China). RAW 264.7 cells were cultured in high-glucose DMEM containing double antibiotics, with 10% fetal bovine serum (FBS) added to the medium. The culture conditions were 37°C in a humidified incubator with 5% CO_2_.

### 3.5. Western Blotting

Proteins were separated by 12% sodium dodecyl sulfate–polyacrylamide gel electrophoresis (SDS-PAGE) and then transferred onto polyvinylidene fluoride (PVDF) membranes. After blocking with 5% non-fat skim milk at room temperature for 45 min, the membranes were washed three times with PBST, followed by incubation with primary antibodies overnight at 4 °C. The membranes were then incubated with horseradish peroxidase (HRP)-conjugated secondary antibodies at room temperature for 45 min, and washed three times with PBST again. Finally, protein bands were visualized using enhanced chemiluminescence (ECL) substrate and captured with a chemiluminescence imaging system.

### 3.6. Aqueous Solubility Study

Solubility was determined by high-performance liquid chromatography (HPLC) according to a previously reported method [41,42]. A stock solution of **15** was prepared and diluted to concentrations ranging from 0.23 to 500 μg/mL to establish a standard curve of peak area versus concentration. An excess amount of **15** was added to 1 mL of purified water to form a saturated solution at 37°C. Following centrifugation at 3000 rpm, the supernatant was analyzed on an HPLC system (Shimadzu LC-20AT, Nakagyo-ku, Japan). The saturated solubility was then calculated using the standard curve equation.

### 3.7. Simulated Gastric and Intestinal Fluid Stability

The SGF and SIF stabilities were measured according to the previously reported method [43]. A solution of **15** (10 μL, 10 mM in DMSO) was added to 990 μL of SGF or SIF. The mixture was incubated at 37 °C. Aliquots (50 μL) were collected at 0, 0.25, 0.5, 1, 2, 4, 8, 10, and 12 h and analyzed by HPLC (Shimadzu LC-20AT). All experiments were performed in triplicate.

### 3.8. Rat Plasma Stability

The stability of **15** in rat plasma was evaluated using a previously reported method [44,45,46]. Specifically, **15** was dissolved in DMSO to prepare a 10 mM stock solution. Then, 4 μL of this compound solution was added to 996 μL of prewarmed rat plasma (maintained at 37 °C) for incubation. At different time points (0, 0.25, 0.5, 1, 2, 4, 8, 10, and 12 h), an equal volume of cold acetonitrile was added to terminate the reaction. After centrifugation, the supernatant was transferred to a new 96-well plate and mixed with purified water (v:v = 1:2). The concentration of **15** was quantified by liquid chromatography–tandem mass spectrometry (LC-MS/MS).

### 3.9. RLM Stability

The stability of **15** in rat liver microsomes was evaluated using a previously reported method [47]. Specifically, **15** was preincubated with rat liver microsomes (RLMs, 0.5 mg/mL) in 100 mM phosphate buffer (pH 7.4) at 37 °C for 5 min. The metabolic reaction was initiated by the addition of 1 mM NADPH. Following incubation at 37 °C for designated time intervals (0, 0.16, 0.25, 0.33, 0.5, 0.75, 1, 1.5, and 2 h), cold acetonitrile was introduced to terminate the reaction and precipitate proteins. After centrifugation, the resulting supernatants were collected and subjected to quantitative analysis via LC-MS/MS.

### 3.10. In Vivo Pharmacokinetics Study

Six healthy male Sprague–Dawley rats (6–8 weeks old, 180 ± 30 g) obtained from GemPharmatech were randomly divided into two groups (*n* = 3 per group). Compound **15** was administered intravenously (3 mg/kg) and orally (10 mg/kg), respectively. Blood samples were collected at designated time points (0.016, 0.083, 0.25, 0.5, 0.75, 1, 2, 4, 6, 8, and 10 h) into heparinized Eppendorf tubes, immediately centrifuged at 12,000 rpm for 5 min at 4 °C, and analyzed by LC-MS/MS (Shimadzu LCMS-8050).

### 3.11. Animal

Animal studies were conducted according to protocols approved by the Institutional Animal Care and Use Committee of China Pharmaceutical University, and the protocol code is YSL-202601049. All animals were used appropriately in a scientifically valid and ethical manner. Male C57BL/6 mice (6–8 weeks old, 18–20 g) (Gempharmatech, Nanjing, China) were acclimatized 3 days prior to experimentation.

### 3.12. DSS-Induced Colitis Model

DSS was dissolved in drinking water to a concentration of 3% (*w*/*v*). C57BL/6 mice were separated into five groups (*n* = 6 per group): (1) control group, (2) DSS model group, (3) DSS + **15** (10 mg/kg) group, (4) DSS + **15** (20 mg/kg) group, (5) DSS + tofacitinib (20 mg/kg) group. Mice were given 3% (*w*/*v*) DSS in drinking water for 7 days to construct the acute colitis model. Compound **15** (10 and 20 mg/kg) and tofacitinib (20 mg/kg) were ground and suspended in DMSO/PEG400/H_2_O (*v*:*v*:*v* = 1:4:5) and administered by gavage daily. Mice were monitored daily for behavioral changes, body weight, and disease activity index (DAI). The detailed criteria for DAI scoring are shown in Table 4. At the end of the experiment, the mice were executed and the colons were collected, measured for their length, and further analyzed.

DAI was calculated using the following formula: DAI score = (weight loss score + fecal trait score + blood in stool score)/3.

### 3.13. IL-6 and TNF-α Production

Levels of IL-6 (EK0410/EK0411, Boster, Wuhan, China) and TNF-α (EK0525/EK0527, Boster, China) were detected using commercially available kits according to the manufacturer’s instructions.

### 3.14. Histopathological Assessment

The resected colonic tissues were fixed in 10% neutral buffered formalin for 24 h. The specimens were further dehydrated and embedded in paraffin. To carry out a histological examination, 4 µm sections of fixed embedded colon tissues were cut and stained with H&E. Pathologists who were unaware of the treatment performed histologic assessments based on a scoring system.

### 3.15. Statistical Analysis

Data are expressed as the mean ± SD. Statistical comparisons were performed using one-way ANOVA with Tukey’s multiple comparisons test in GraphPad Prism 8.0. Statistical significance was considered at *p* < 0.05.

## 4. Conclusions

In summary, a series of novel pyrrolo [2,3-*b*] pyridine derivatives were developed in this study via a structure-based drug design (SBDD) strategy using tofacitinib as the lead compound. Among them, compound **15** is a potent JAK1 inhibitor with an IC_50_ value of 0.48 nM. Molecular docking results demonstrated that the alkynylpyridine moiety in compound **15** forms a stable hydrogen bond with the Arg879 residue, and its molecular structure exhibits better complementarity to the ATP-binding pocket of JAK1 protein. Consequently, compound **15** shows significantly enhanced JAK1 inhibitory activity compared to tofacitinib. At the cellular level, compound **15** exhibited favorable inhibitory activity against the JAK/STAT signaling pathway. Furthermore, compound **15** displayed significantly improved pharmacokinetic properties, including excellent in vitro stability and an oral bioavailability of 34% in rats. In the DSS-induced acute colitis model, compound **15** significantly reduced the DAI score, inhibited the production of pro-inflammatory cytokines (TNF-α and IL-6), and ameliorated histopathological features. Although short-term in vivo administration observations reveal good tolerability of this compound at therapeutic doses, comprehensive safety evaluations including acute and chronic toxicity tests, cardiovascular safety assays, and dedicated investigations into class-wide risks such as immunosuppression and increased infection susceptibility of JAK inhibitors have not been performed in the present study. In subsequent research, we will conduct a full panel of preclinical safety assessments in a phased manner to fully characterize the safety profile of compound 15. Overall, compound 15, as a novel and potent JAK1 inhibitor, possesses promising potential for the treatment of inflammatory bowel disease.

## Figures and Tables

**Table 1:** Structures and enzymatic potencies for compounds **9**–**15**.

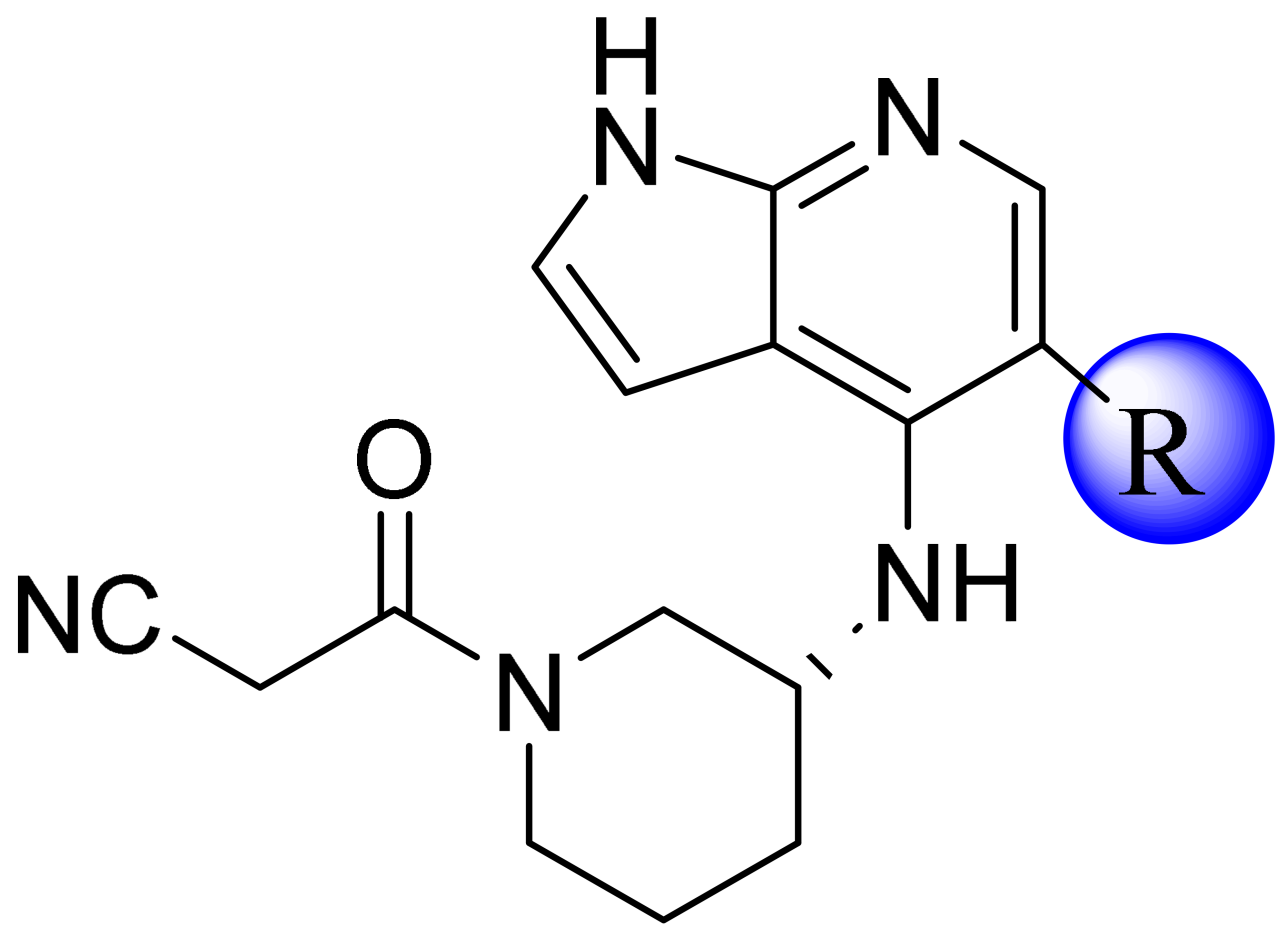		
Compd.	R	JAK1 IC_50_ (nM) *^a^*
**9**	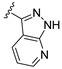	3.532 ± 0.190
**10**	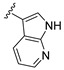	36.5 ± 3.694
**11**	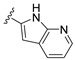	90.07 ± 5.940
**12**	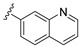	48.78 ± 0.799
**13**	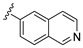	47.72 ± 6.074
**14**	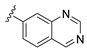	39.64 ± 2.687
**15**	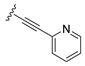	0.48 ± 0.427
**Tofaticinib**	/	2.66 ± 0.359

*^a^* Measured at 2 μM ATP concentration. Compounds were assayed at least twice and the IC_50_ was reported as the geometric mean.

**Table 2:** Structures and enzymatic potencies for compounds **16**–**18**.

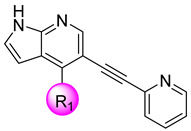		
Compd.	R_1_	JAK1 IC_50_ (nM) *^a^*
**16**	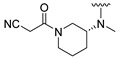	10.54 ± 1.548
**17**	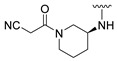	61.74 ± 10.720
**18**	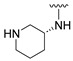	23.27 ± 1.209
**Tofaticinib**	/	2.66 ± 0.359

*^a^* Measured at 2 μM ATP concentration. Compounds were assayed at least twice and the IC_50_ was reported as the geometric mean.

**Table 3:** Solubility ^a^ of **15** in water, stability in SIF ^b^, SGF ^b^, rat plasma ^b^ and metabolic stability in RLM in vitro.

Parameters	15	Parameters	15
Solubility (μg/mL)	462.34	RLM T_1/2_ (min)	131.20 ± 1.90
SIF	94.67	RLM Cl_int_ (μL/min/mg)	5.28 ± 0.08
SGF	96.24	RLM 30 min remaining (%)	81.28 ± 2.99
Rat plasma	85.91	-	-

^a^ Solubility testing by HPLC method. ^b^ Remaining (%) after 12 h of incubation.

**Table 4:** Scoring criteria for DAI.

Scores	Weight Loss (%)	Fecal Trait	Blood in Stool
0	0	normal	normal
1	<5	soft stool	occult blood positive
2	5–10	pasty stool	slight visible blood
3	10–15	watery stool	obvious hematochezia
4	>15	liquid stool	gross bloody stool

## Data Availability

The original contributions presented in this study are included in the article/Supplementary Material. Further inquiries can be directed to the corresponding authors.

## Supplementary Materials

The following supporting information can be downloaded at https://www.mdpi.com/article/10.3390/molecules31132236/s1. Supplementary information contains the detection of raw data of Western blot, and structure characterization data of all target compounds.

## Abbreviations

IBD, inflammatory bowel disease; CD, Crohn’s disease; UC, ulcerative colitis; DAI, disease activity index; DSS, dextran sulfate sodium; ELISA, enzyme-linked immunosorbent assay; H&E, hematoxylin and eosin; SAR, structure−activity relationship; WB, Western blot; SGF, simulated gastric fluid; SIF, simulated intestinal fluid; RLM, rat liver microsomes.
