# Supplementary material for: Identification of Pyrrolo [2,3-b] Pyridine Derivatives as Novel JAK1 Inhibitors for the Treatment of Inflammatory Bowel Disease
Source: Molecules. 2026 Jun 25;31(13):2236. doi: 10.3390/molecules31132236 (PMC13362992; doi:10.3390/molecules31132236)

Supplementary Information

Identiffcation of Pyrrolo [2,3-b] Pyridin Derivatives as Novel JAK1 Inhibitors for the Treatment of Inflammatory Bowel Disease

Shuai Wen [a, b], Can Xiao [b], Li-min Du [b], Jing Ji [a], Hong-kai Sha [a], Shun-xin Wang Lin [c]， Yi Mou [a,*], Hao Sun [c,*], and Zheng-yu Jiang [b,*]

[a] College of Pharmacy, Taizhou University, Taizhou 225300, China; wen15298503462@126.com (S.W.); jijing@jou.edu.cn (J.J.); shahongkaitzxy@163.com (H.-K.S.); mouyicpu@163.com (Y. M.)

[b] Jiangsu Key Laboratory of Drug Design and Optimization, China Pharmaceutical University, Nanjing 210009, China; asd789456123aa@163.com (C. X.); dminmin@163.com (L.-M.D.); jiangzhengyucpu@163.com (Z.-Y.J.)

[c] Nanjing Linkinovo Biological Technology Research Institute, Nanjing 211100, China; shun@linkinovo.com.cn (S.-X.WL.); sunh@comtruelab.com (H. S.)

[*]Corresponding author. E-mail addresses: mouyicpu@163.com (Y. M.); sunh@comtruelab.com (H. S.); jiangzhengyucpu@163.com (Z.-Y.J.)

Raw data of western blot.

Raw data of western blot of Fig. 5

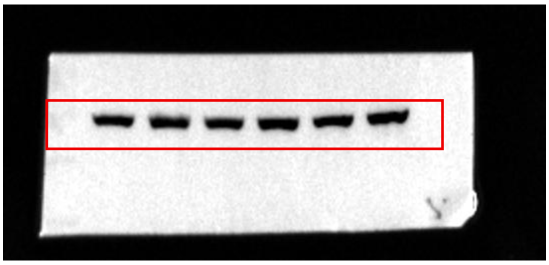

Actin 42kDa

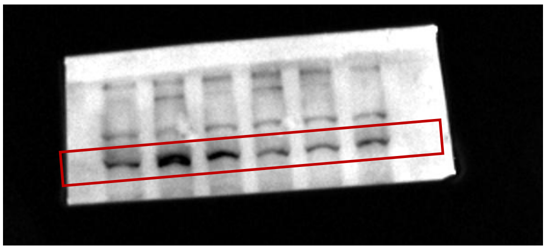

P-STAT1 91kDa

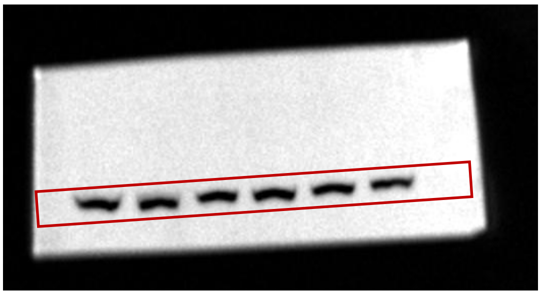

STAT1 91kDa

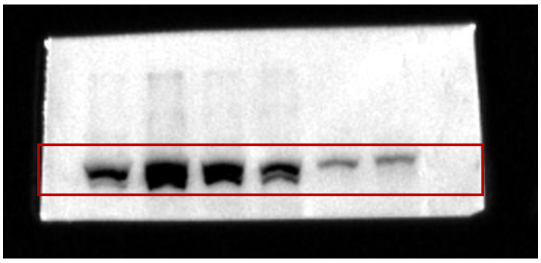

P-STAT3 88kDa

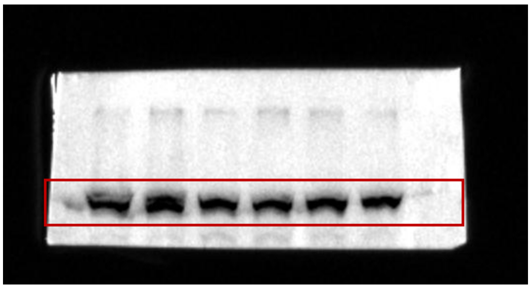

STAT3 88kDa

Structure characterization of compounds 9-18.

^{1}H NMR (300 MHz, DMSO-d_6), ^{13}C NMR (75 MHz, DMSO-d_6), HRMS, and HPLC for **9**

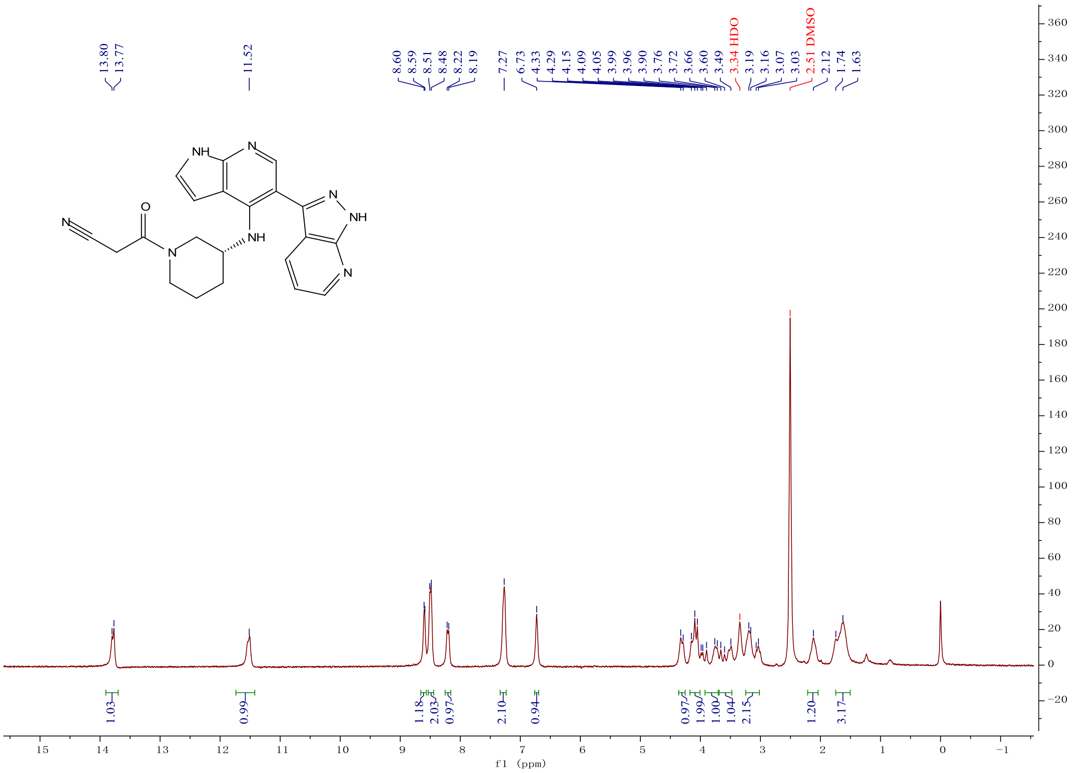

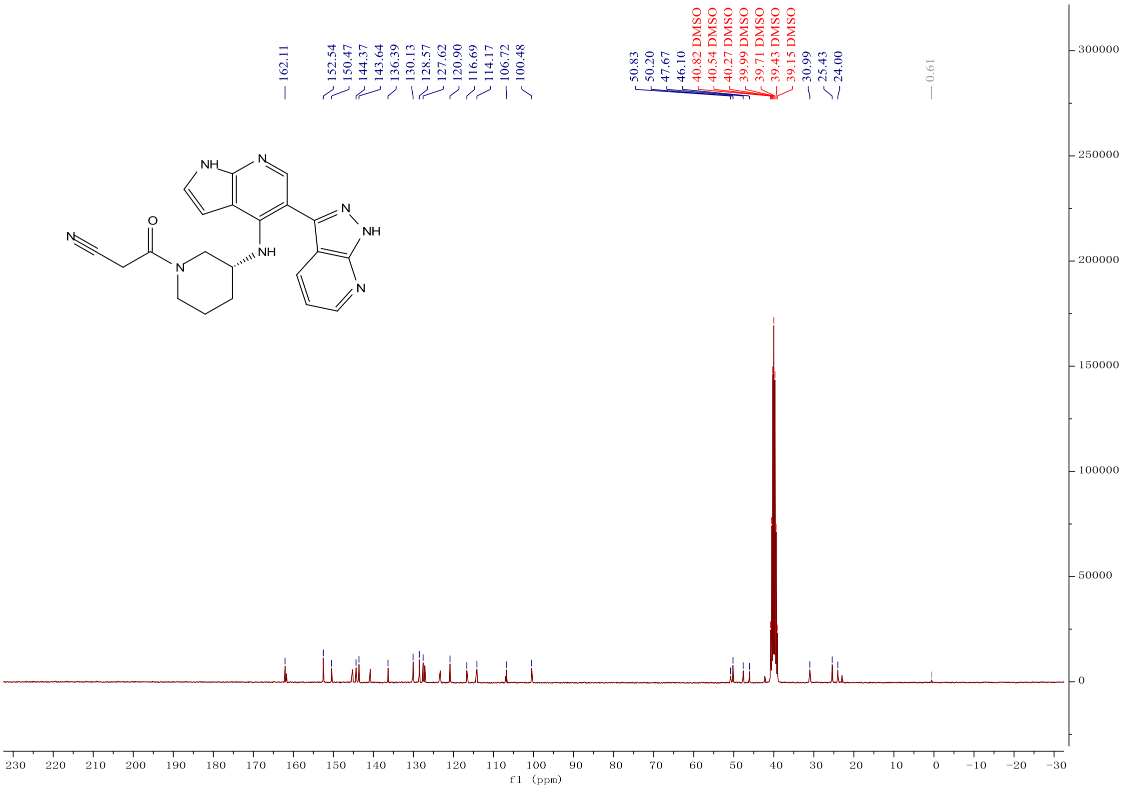

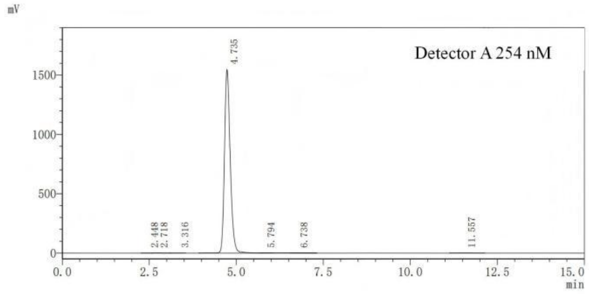

<Peak Table>
Detector A 254 nM

Number	Retention time	Area	Height	Area%
1	2.448	1217	118	0.007
2	2.718	5861	457	0.033
3	3.316	1390	139	0.008
4	4.735	17756909	1544972	99.859
5	5.794	2850	283	0.016
6	6.738	5243	270	0.029
7	11.557	8479	319	0.048
Total		17781948	1546557	100.000

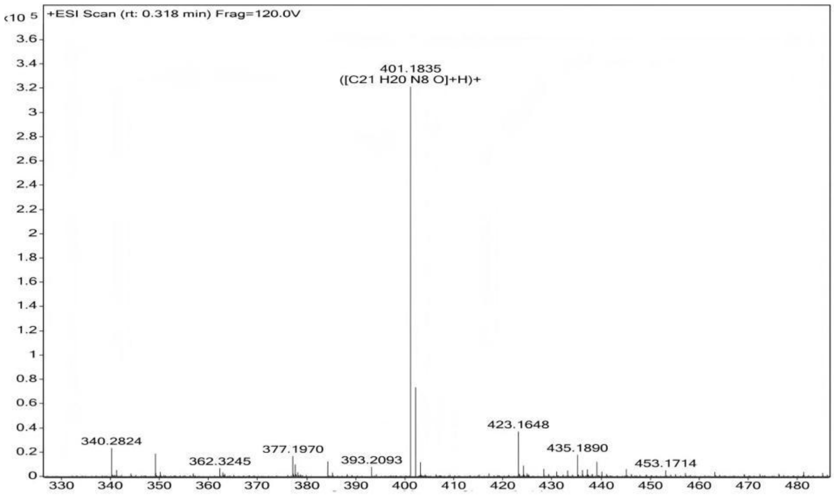

^{1}H NMR (300 MHz, DMSO-d_6), ^{13}C NMR (75 MHz, DMSO-d_6), HRMS, and HPLC for **10**

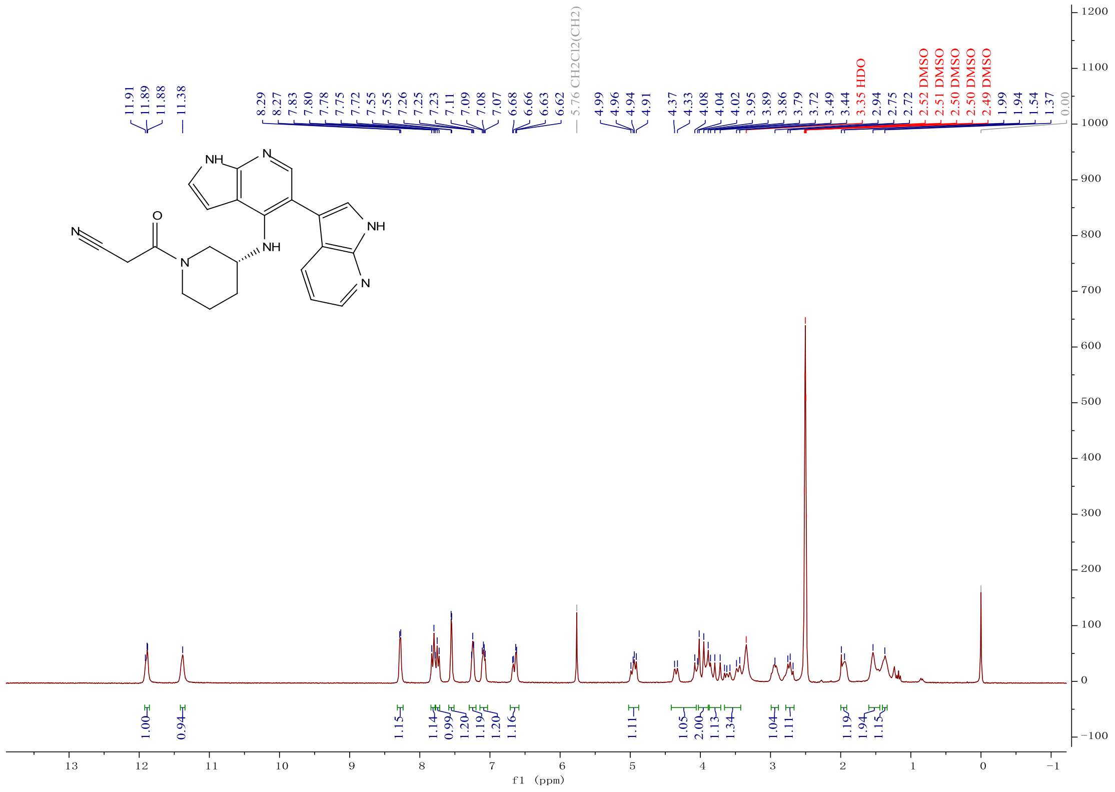

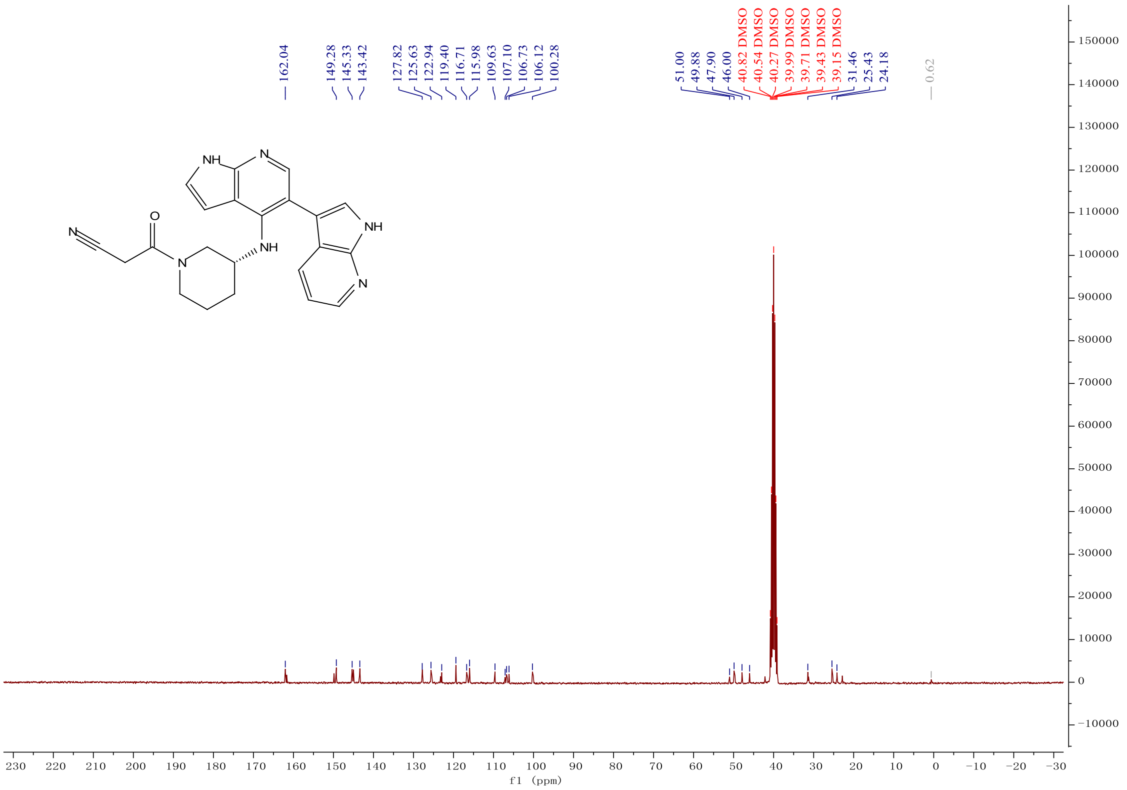

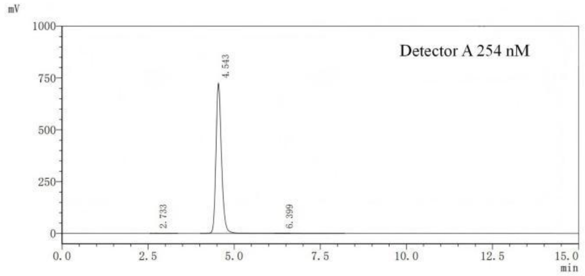

<Peak Table>
Detector A 254 nM

Number	Retention time	Area	Height	Area%
1	2.733	8146	564	0.099
2	4.543	8184242	724430	99.763
3	6.399	11284	663	0.138
Total		8203672	725657	100.000

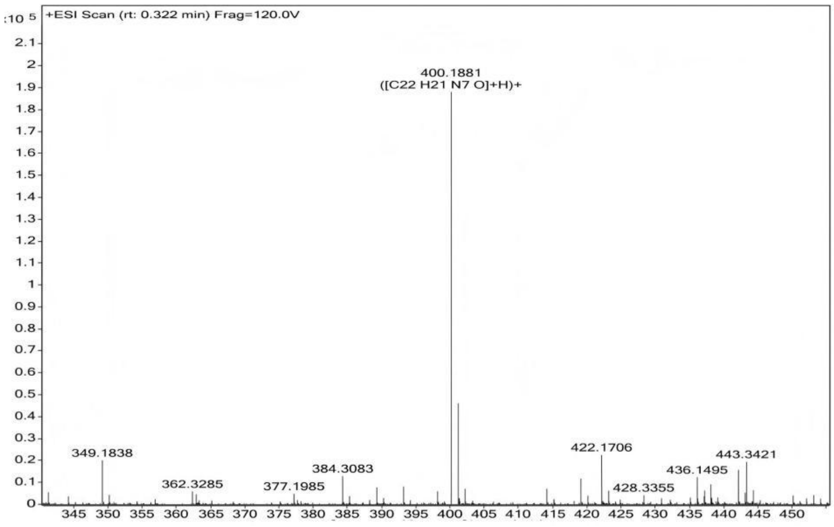

^{1}H NMR (300 MHz, DMSO-d_6), ^{13}C NMR (75 MHz, DMSO-d_6), HRMS, and HPLC for **11**

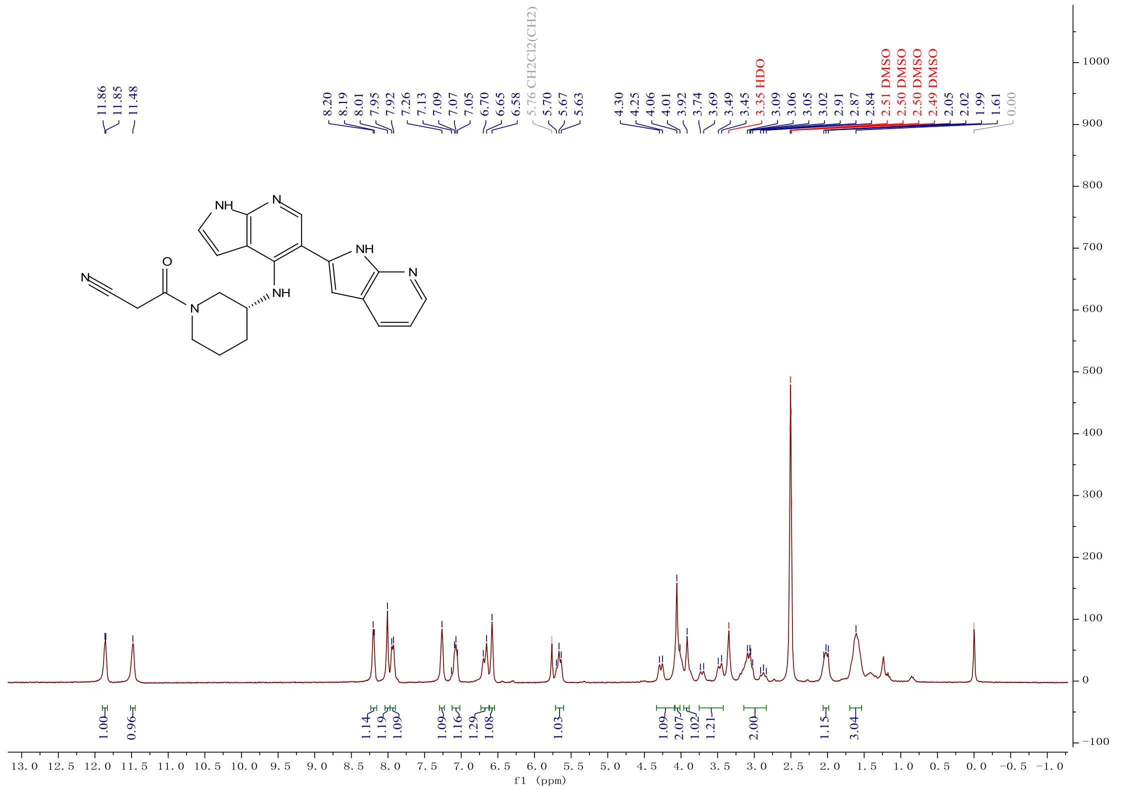
11.86
11.85
11.48
8.20
8.19
8.01
7.95
7.92
7.26
7.13
7.09
7.07
7.05
6.70
6.65
6.58
5.76 CH2Cl2(CH2)
5.70
5.67
5.63
4.30
4.25
4.06
4.01
3.92
3.74
3.69
3.49
3.45
3.35 HDO
3.09
3.06
3.05
3.02
2.91
2.87
2.84
2.51 DMSO
2.50 DMSO
2.50 DMSO
2.49 DMSO
2.05
2.02
1.99
1.61
0.00
NH
N
NH
N
O
N
N
NH
1.00
0.96
1.14
1.19
1.09
1.09
1.16
1.29
1.08
1.03
1.09
2.07
1.02
1.21
2.00
1.15
3.04
f1 (ppm)

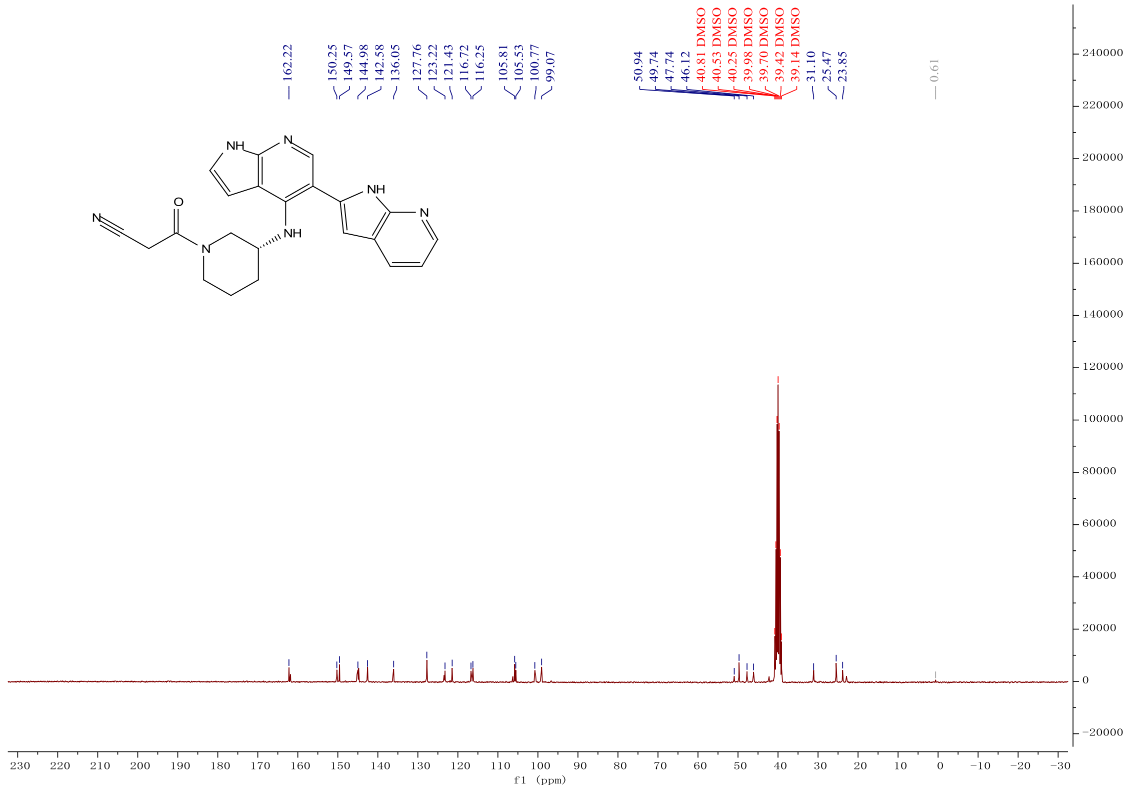
162.22
150.25
149.57
144.98
142.58
136.05
127.76
123.22
121.43
116.72
116.25
105.81
105.53
100.77
99.07
50.94
49.74
47.74
46.12
40.81 DMSO
40.53 DMSO
40.25 DMSO
39.98 DMSO
39.70 DMSO
39.42 DMSO
39.14 DMSO
31.10
25.47
23.85
0.61
NH
N
NH
N
O
N
N
NH
f1 (ppm)

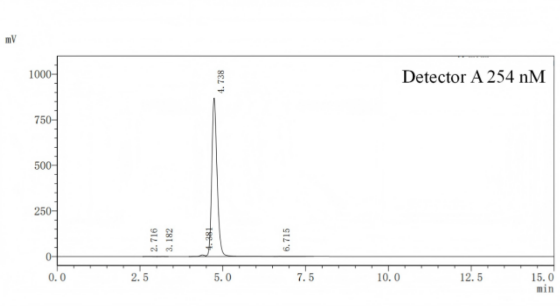

<Peak Table>
Detector A 254 nM

Number	Retention time	Area	Height	Area%
1	2.716	2872	209	0.029
2	3.182	1318	127	0.013
3	4.381	80928	7973	0.816
4	4.738	9819056	869826	99.065
5	6.715	7551	313	0.076
Total		9911725	878449	100.000

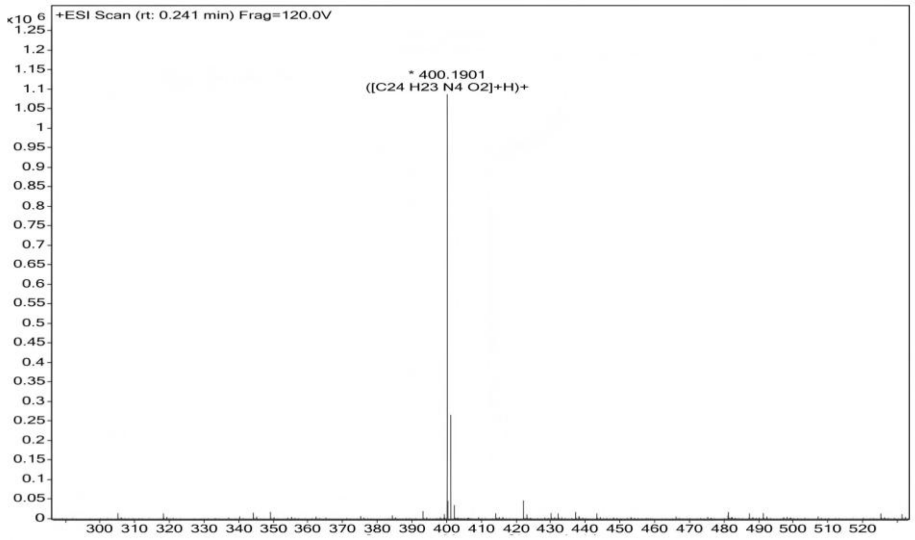

^{1}H NMR (300 MHz, DMSO-d_6), ^{13}C NMR (75 MHz, DMSO-d_6), HRMS, and HPLC for **12**

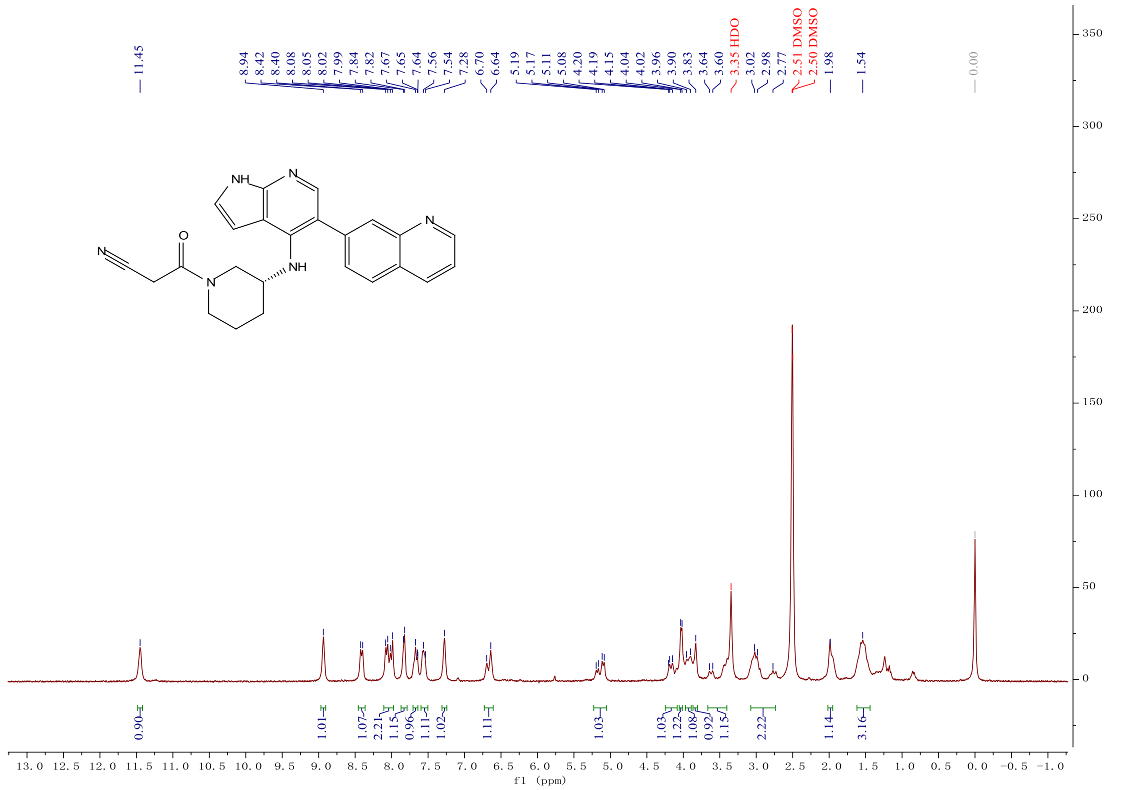

11.45
8.94
8.42
8.40
8.08
8.05
8.02
7.99
7.84
7.82
7.67
7.65
7.64
7.56
7.54
7.28
6.70
6.64
5.19
5.17
5.11
5.08
4.20
4.19
4.15
4.04
4.02
3.96
3.90
3.83
3.64
3.60
3.35 HDO
3.02
2.98
2.77
2.51 DMSO
2.50 DMSO
1.98
1.54
0.00
NH
N
N
O
N
NH
N
0.90
1.01
1.07
2.21
1.15
0.96
1.11
1.02
1.11
1.03
1.03
1.22
1.08
0.92
1.15
2.22
1.14
3.16
f1 (ppm)

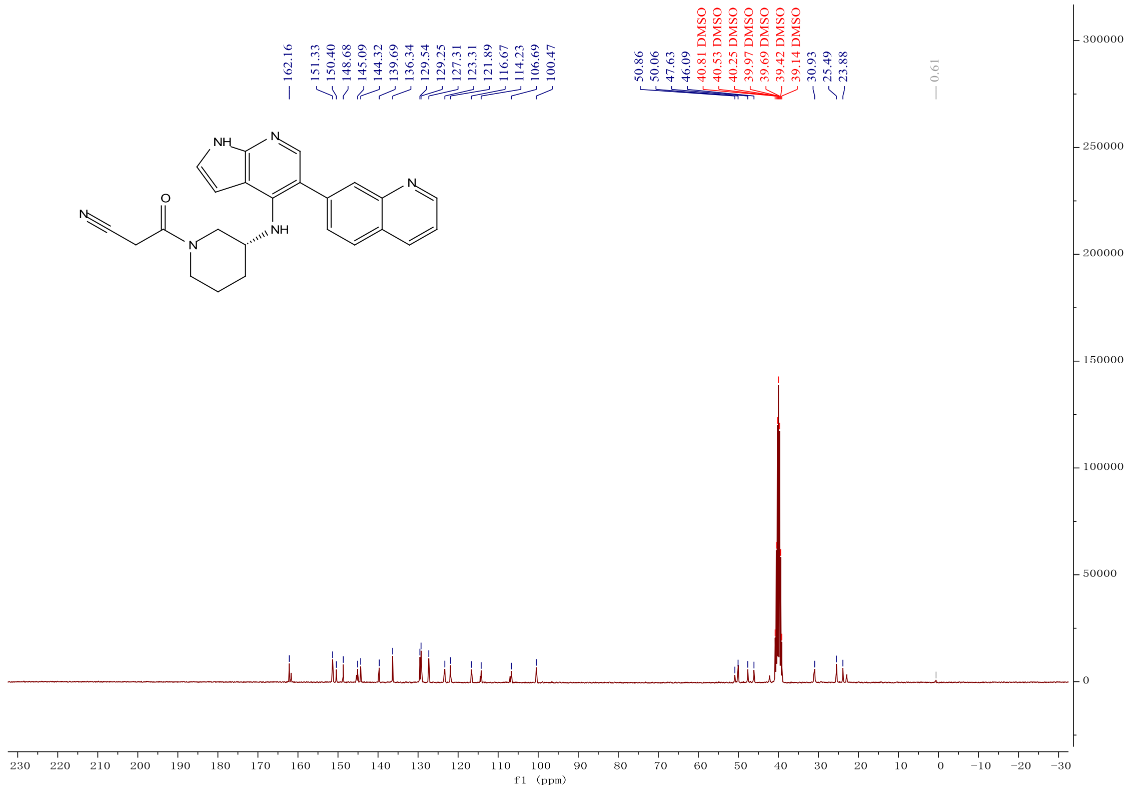

162.16
151.33
150.40
148.68
145.09
144.32
139.69
136.34
129.54
129.25
127.31
123.31
121.89
116.67
114.23
106.69
100.47
50.86
50.06
47.63
46.09
40.81 DMSO
40.53 DMSO
40.25 DMSO
39.97 DMSO
39.69 DMSO
39.42 DMSO
39.14 DMSO
30.93
25.49
23.88
0.61
NH
N
N
O
N
NH
N
f1 (ppm)

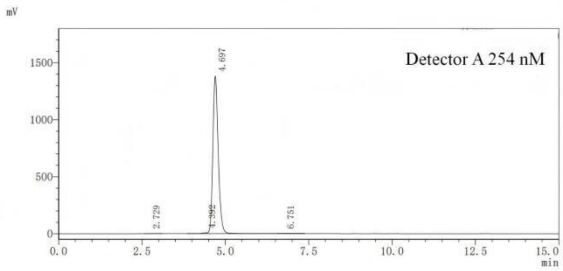

<Peak Table>
Detector A 254 nM

Number	Retention time	Area	Height	Area%
1	2.729	4310	312	0.028
2	4.392	61163	7135	0.013
3	4.697	15538926	1381513	99.546
4	6.751	5399	283	0.035
Total		15609797	1389243	100.000

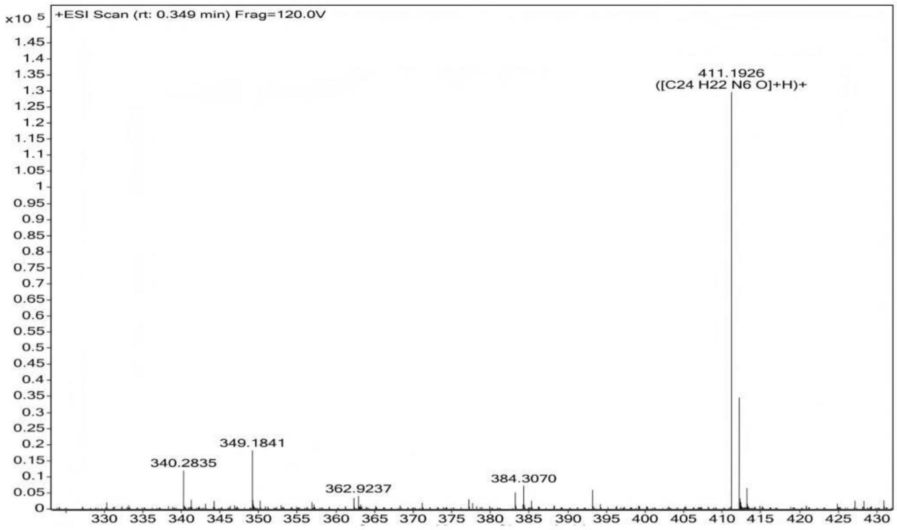

^{1}H NMR (300 MHz, DMSO-d_6), ^{13}C NMR (75 MHz, DMSO-d_6), HRMS, and HPLC for **13**

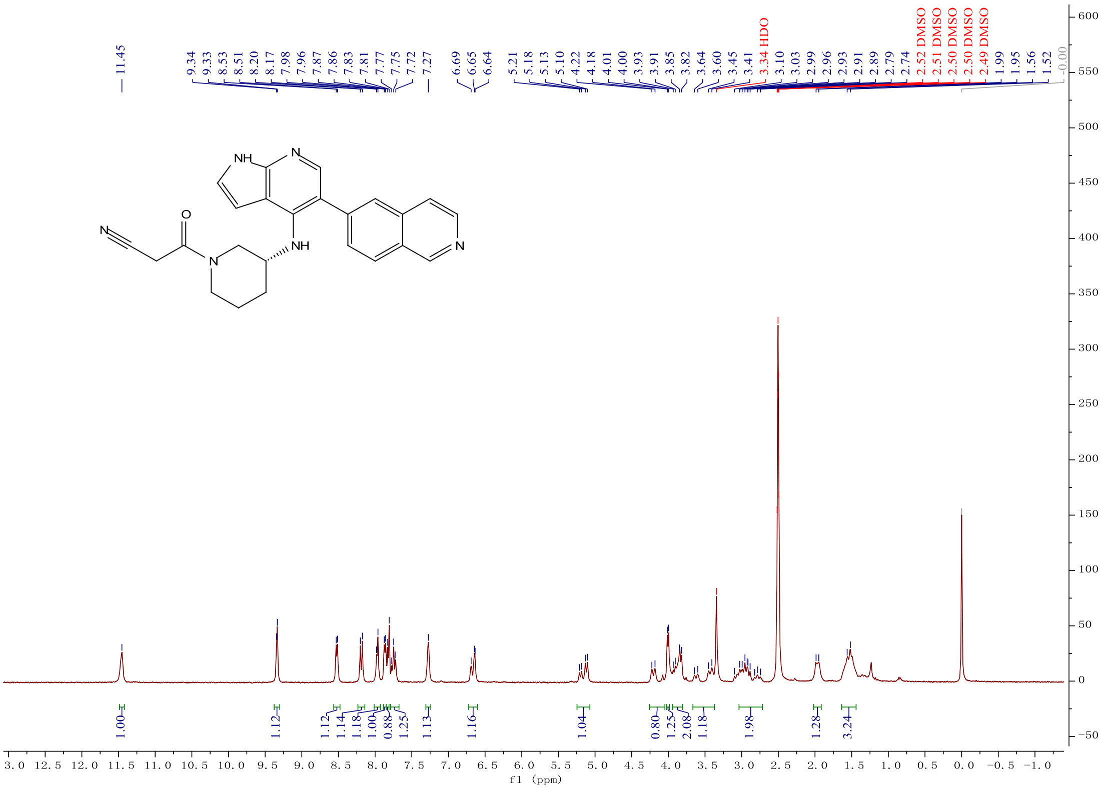

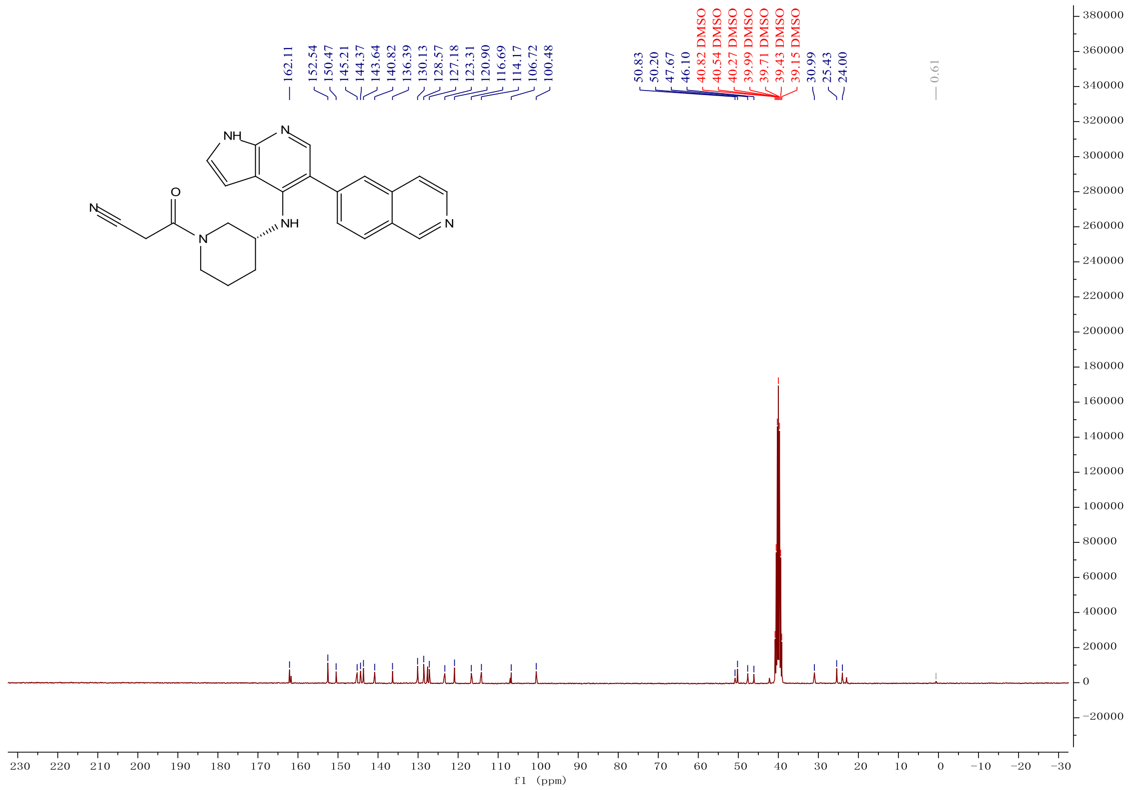

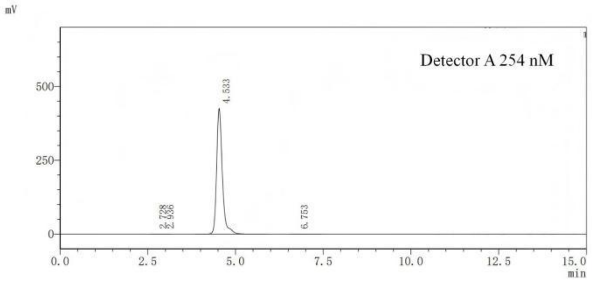

<Peak Table>
Detector A 254 nM

Number	Retention time	Area	Height	Area%
1	2.728	2175	200	0.045
2	2.936	1383	138	0.028
3	4.533	4845574	425339	99.779
4	6.753	7150	297	0.147
Total		4856282	425974	100.000

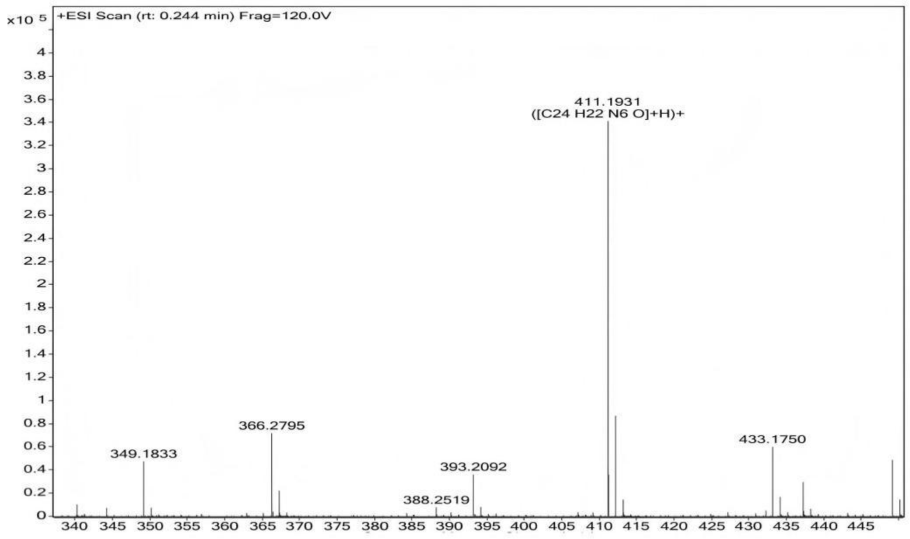

^{1}H NMR (300 MHz, DMSO-d_6), ^{13}C NMR (75 MHz, DMSO-d_6), HRMS, and HPLC for **14**

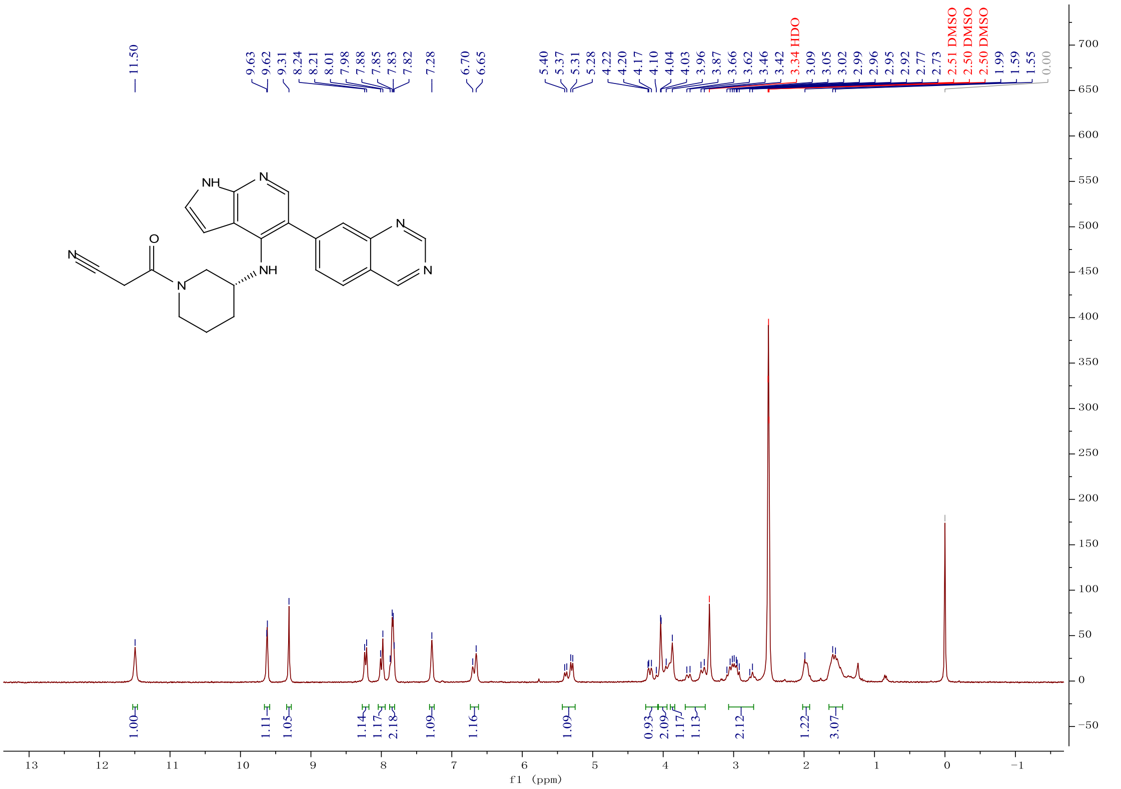
11.50
9.63
9.62
9.31
8.24
8.21
8.01
7.98
7.88
7.85
7.83
7.82
7.28
6.70
6.65
5.40
5.37
5.31
5.28
4.22
4.20
4.17
4.10
4.04
4.03
3.96
3.87
3.66
3.62
3.46
3.42
3.34 HDO
3.09
3.05
3.02
2.99
2.96
2.95
2.92
2.77
2.73
2.51 DMSO
2.50 DMSO
2.50 DMSO
1.99
1.59
1.55
0.00
NH
N
O
N
NH
N
N
N
1.00
1.11
1.05
1.14
1.17
2.18
1.09
1.16
1.09
0.93
2.09
1.17
1.13
2.12
1.22
3.07
f1 (ppm)

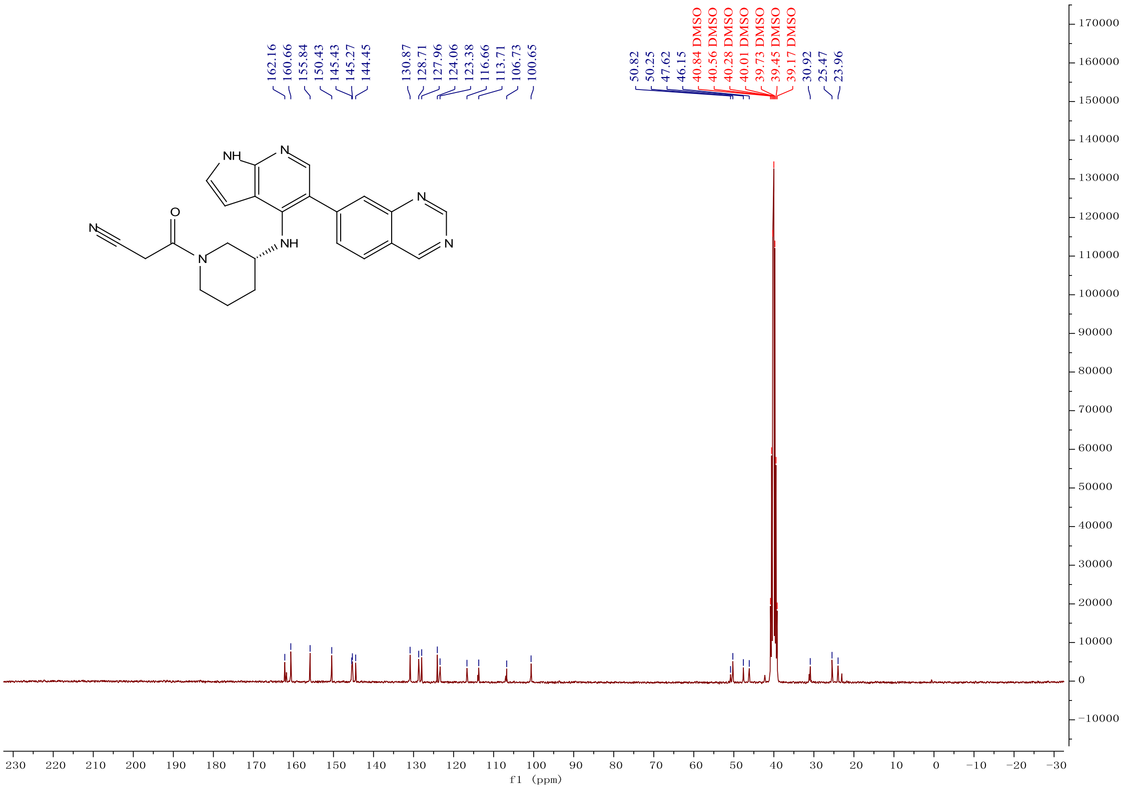
162.16
160.66
155.84
150.43
145.43
145.27
144.45
130.87
128.71
127.96
124.06
123.38
116.66
113.71
106.73
100.65
50.82
50.25
47.62
46.15
40.84 DMSO
40.56 DMSO
40.28 DMSO
40.01 DMSO
39.73 DMSO
39.45 DMSO
39.17 DMSO
30.92
25.47
23.96
NH
N
O
N
NH
N
N
N
f1 (ppm)

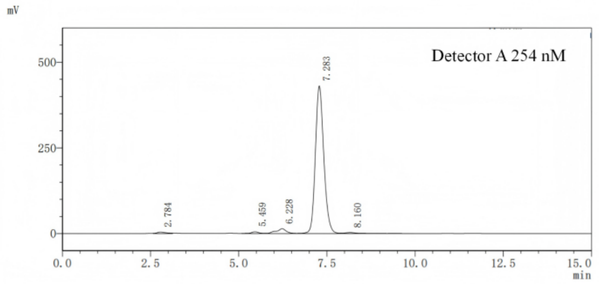

<Peak Table>
Detector A 254 nM

Number	Retention time	Area	Height	Area%
1	2.784	78320	4295	1.017
2	5.459	81384	5248	1.056
3	6.228	288879	14229	3.750
4	7.283	7178517	430848	93.175
5	8.160	77219	2851	1.002
Total		7704319	457470	100.000

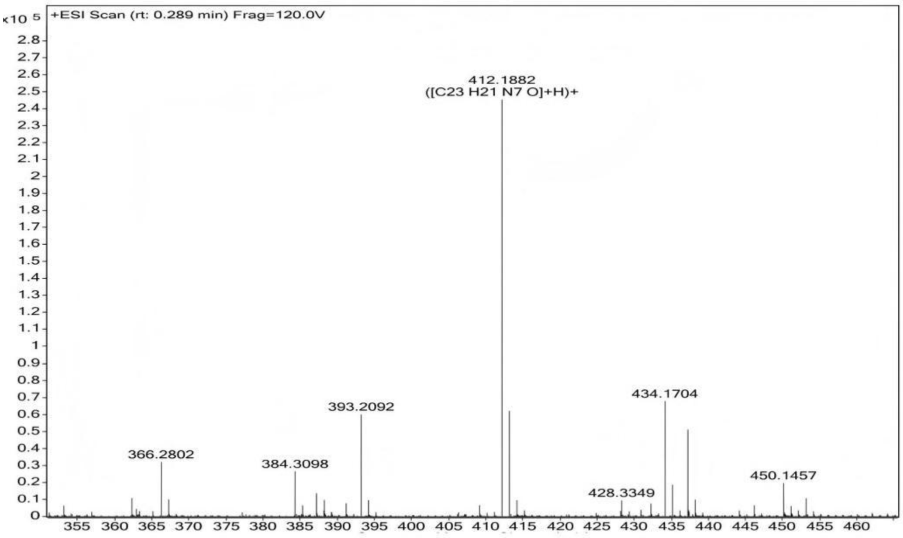

^{1}H NMR (300 MHz, DMSO-d_6), ^{13}C NMR (75 MHz, DMSO-d_6), HRMS, and HPLC for **15**

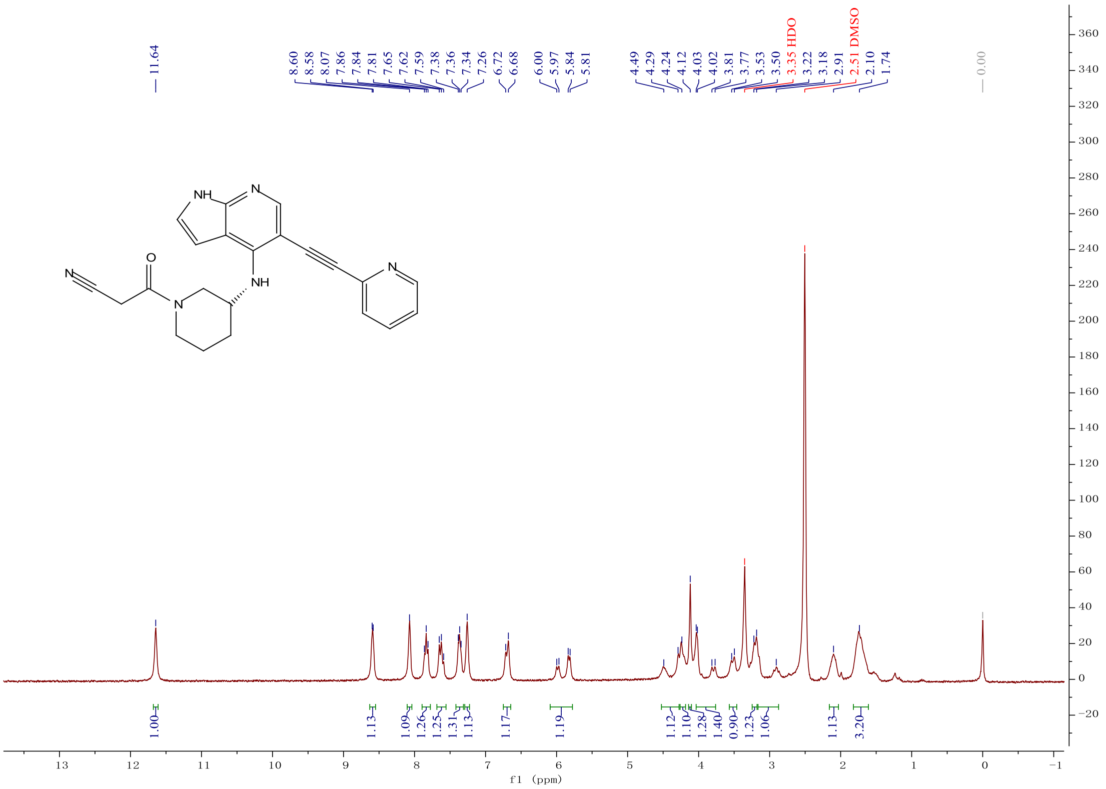

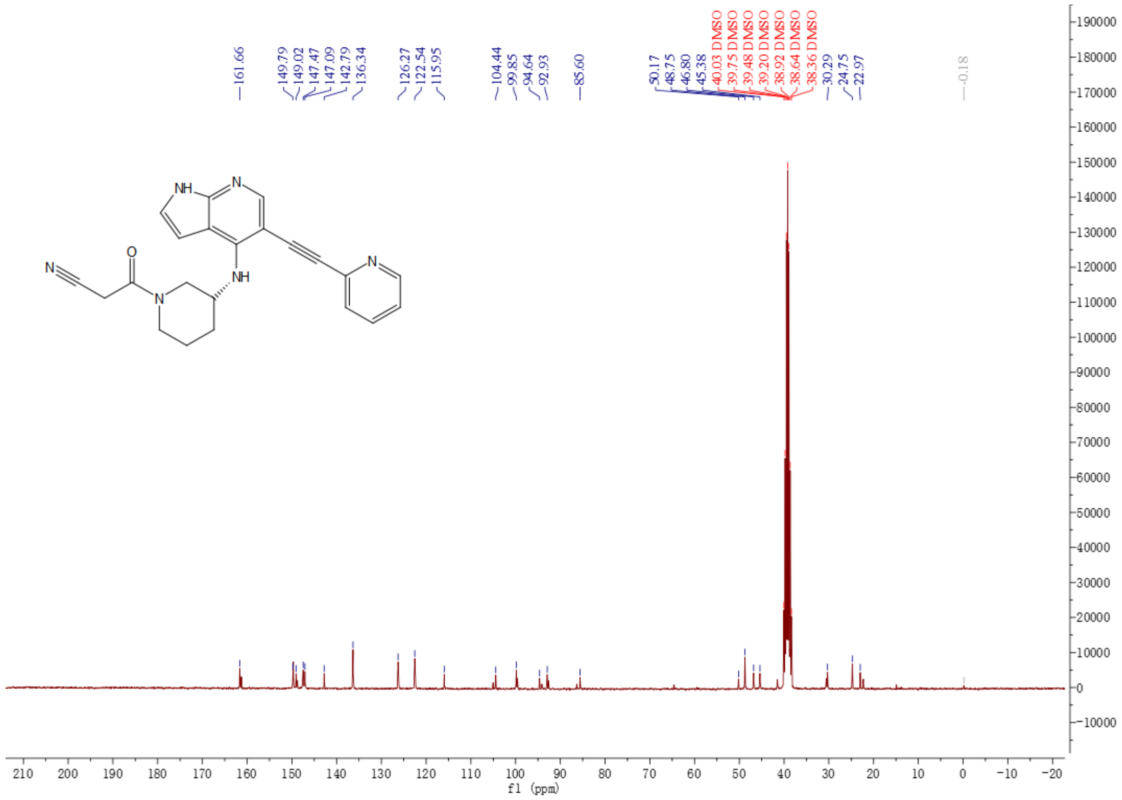

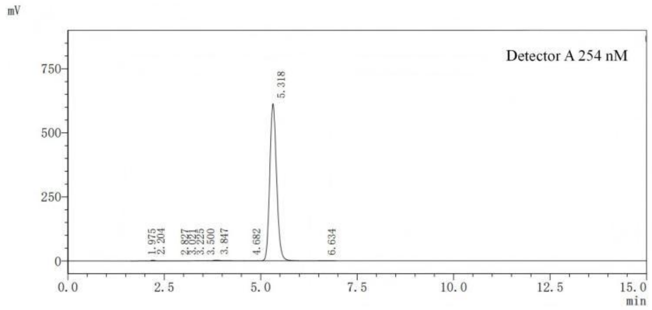

<Peak Table>
Detector A 254 nM

Number	Retention time	Area	Height	Area%
1	1.975	2832	196	0.037
2	2.204	21664	3110	0.283
3	2.827	5003	294	0.065
4	3.021	1060	137	0.014
5	3.225	1957	138	0.026
6	3.500	1810	143	0.024
7	3.847	48698	3075	0.635
8	4.682	2514	183	0.033
9	5.318	7580182	612995	98.855
10	6.634	2224	159	0.029
Total		7667944	620430	100.000

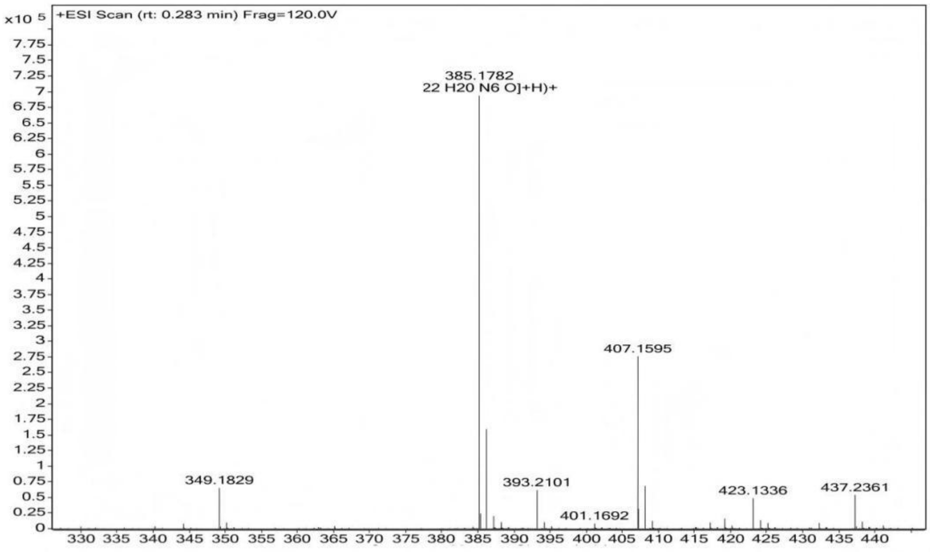

^{1}H NMR (300 MHz, DMSO-d_6), HRMS, and HPLC for **16**

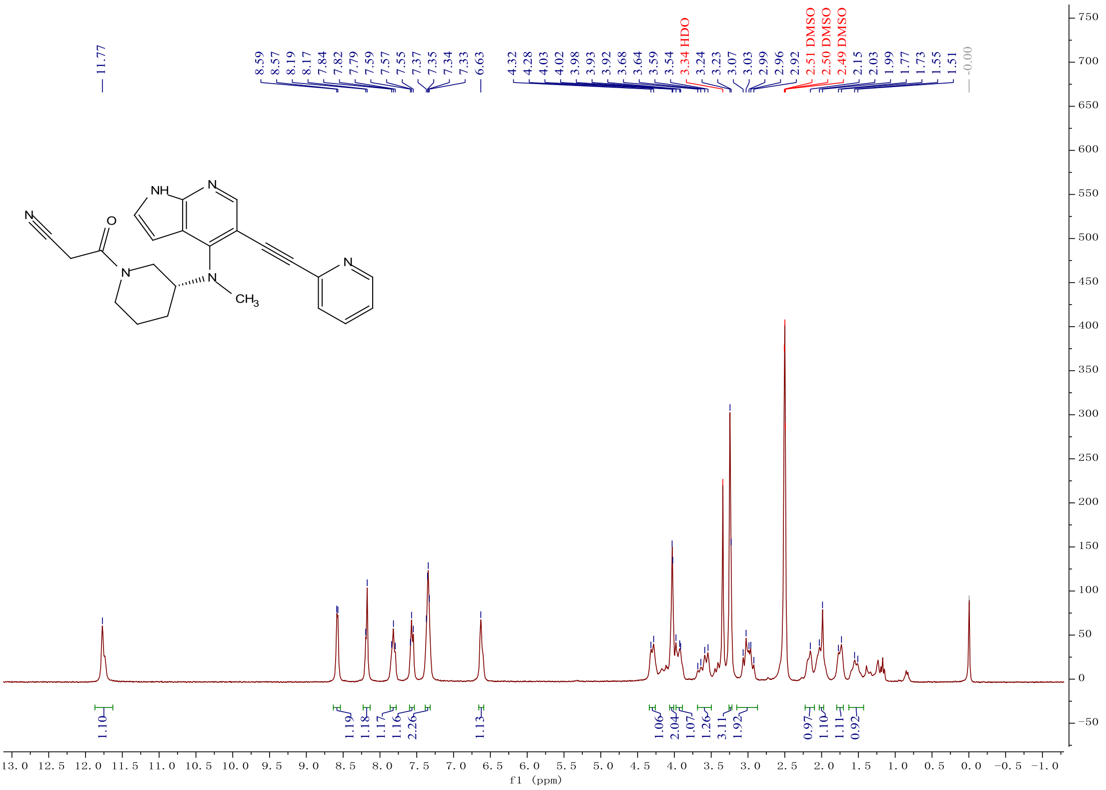

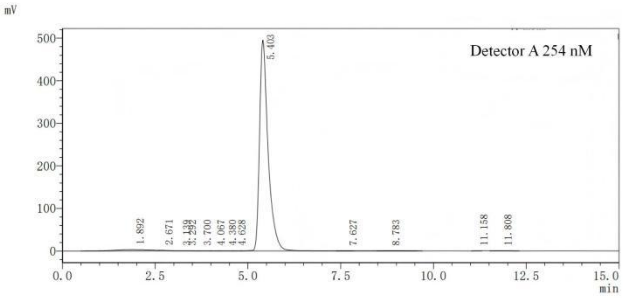

<Peak Table>
Detector A 254 nM

Number	Retention time	Area	Height	Area%
1	1.892	207320	2960	2.507
2	2.671	33478	1741	0.405
3	3.139	5725	528	0.069
4	3.292	9319	506	0.113
5	3.700	4030	252	0.049
6	4.067	1982	158	0.024
7	4.380	3337	185	0.040
8	4.628	1571	127	0.019
9	5.403	7985396	494767	96.546
10	7.627	1318	81	0.016
11	8.783	11465	425	0.139
12	11.158	1656	106	0.020
13	11.808	4506	149	0.054
Total		8271103	501984	100.000

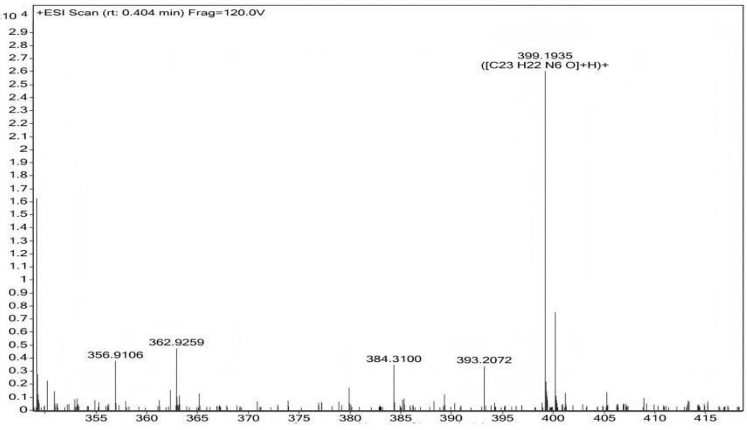

^{1}H NMR (300 MHz, DMSO-d_6), HRMS, and HPLC for **17**

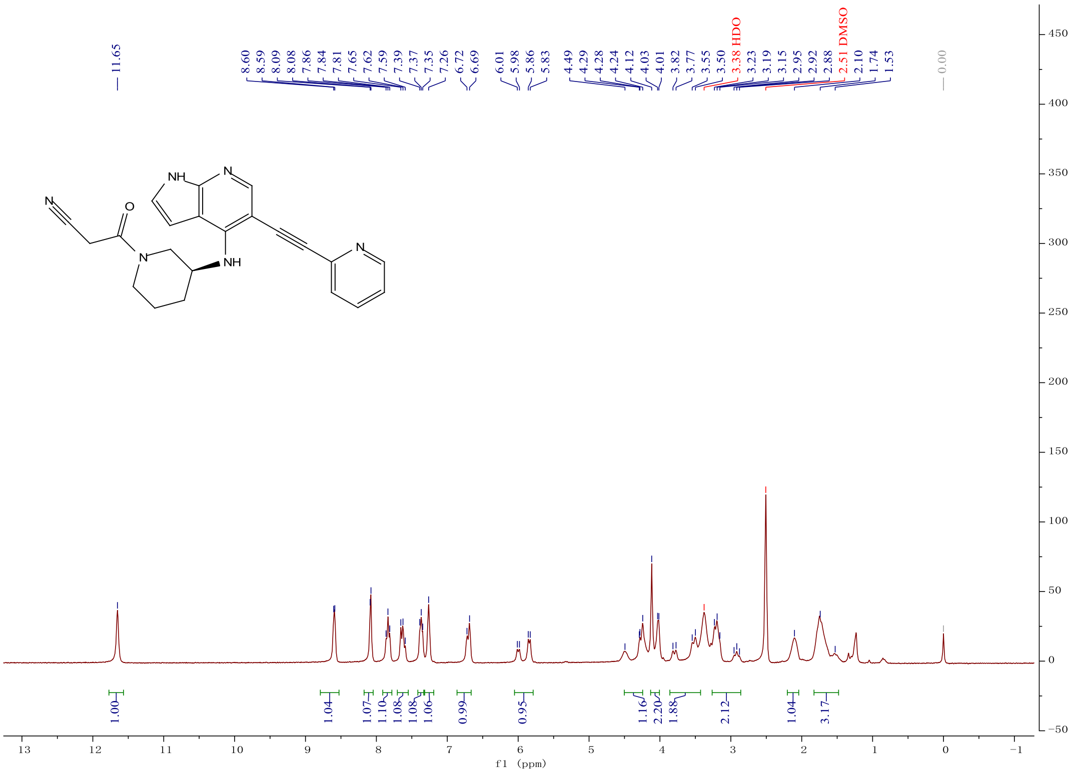

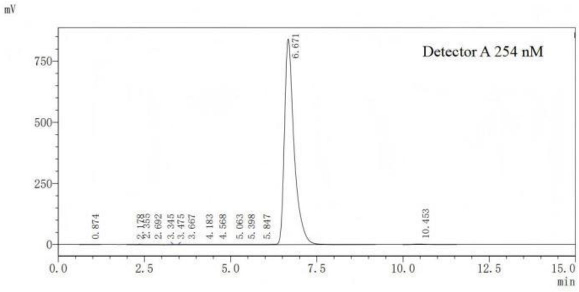

<Peak Table>
Detector A 254 nM

Number	Retention time	Area	Height	Area%
1	0.874	1574	66	0.010
2	2.178	5402	750	0.033
3	2.355	15567	1944	0.094
4	2.692	7252	511	0.044
5	3.345	4289	283	0.026
6	3.475	1098	248	0.007
7	3.667	15182	902	0.092
8	4.183	1515	175	0.009
9	4.568	4267	186	0.026
10	5.063	9420	560	0.057
11	5.398	8246	553	0.050
12	5.847	4555	242	0.028
13	6.671	16395625	840180	99.145
14	10.453	63104	2210	0.382
Total		16537096	848811	100.000

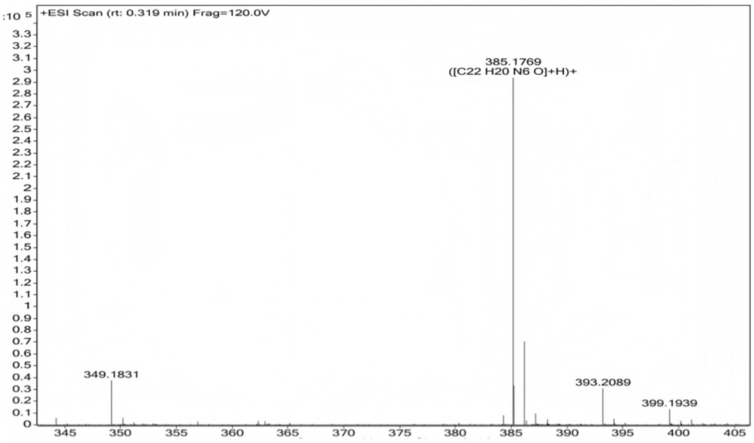

^{1}H NMR (300 MHz, DMSO-d_6), HRMS, and HPLC for **18**

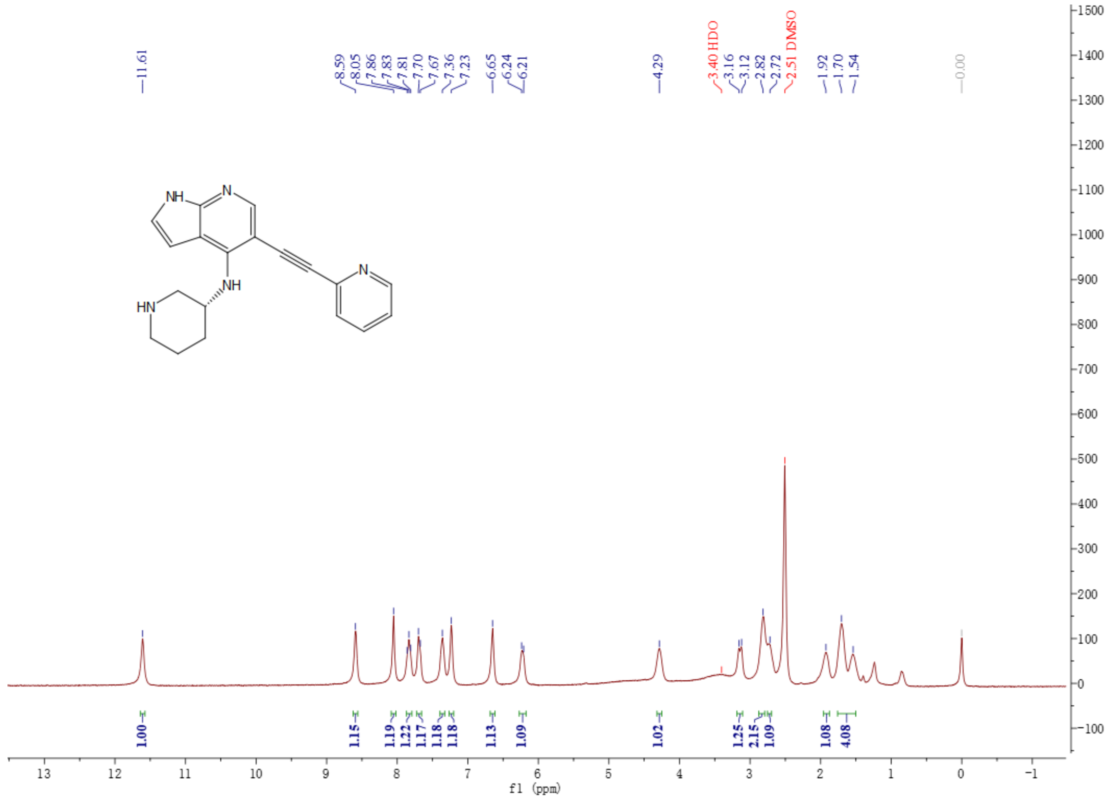

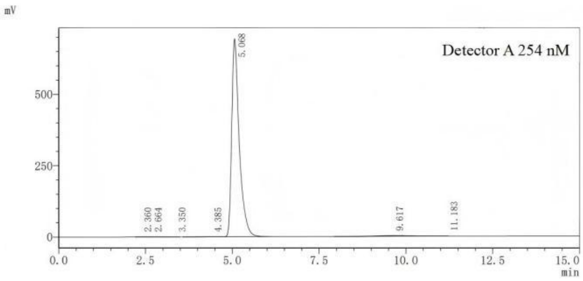

<Peak Table>
Detector A 254 nM

Number	Retention time	Area	Height	Area%
1	2. 360	8349	1002	0. 078
2	2. 664	5804	394	0. 054
3	3. 350	1134	71	0. 011
4	4. 385	22865	781	0. 213
5	5. 068	10504221	693422	98. 004
6	9. 617	174361	2167	1. 627
7	11. 183	1425	184	0. 013
Total		10718159	698022	100.000

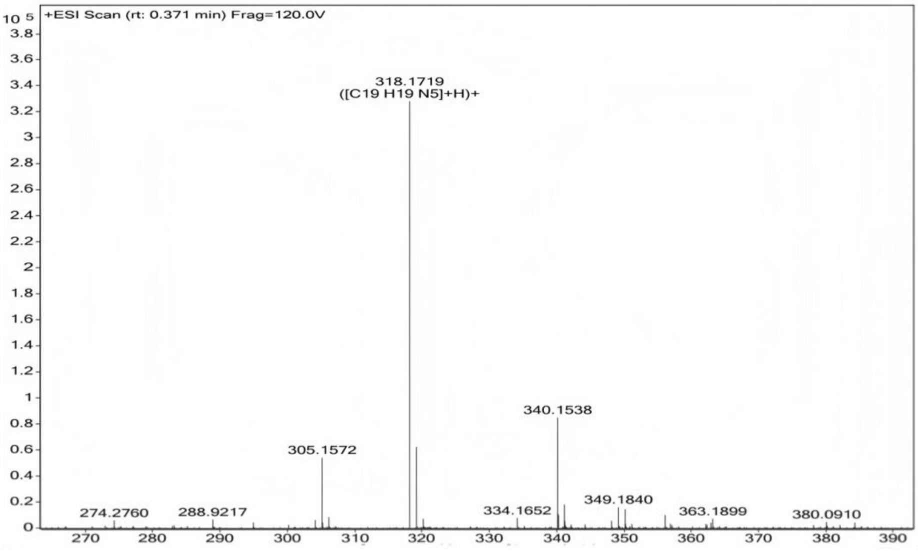

Supplement: Supplementary file 1 [file molecules-31-02236-s001.zip › molecules-4374387-supplementary.pdf]
